# Vitamin Status in Patients with Phenylketonuria: A Systematic Review and Meta-Analysis

**DOI:** 10.3390/ijms25105065

**Published:** 2024-05-07

**Authors:** Kamila Bokayeva, Małgorzata Jamka, Dariusz Walkowiak, Monika Duś-Żuchowska, Karl-Heinz Herzig, Jarosław Walkowiak

**Affiliations:** 1 Department of Pediatric Gastroenterology and Metabolic Diseases, Poznan University of Medical Sciences, Szpitalna Str. 27/33, 60-572 Poznań, Poland; kamila.bokayeva@student.ump.edu.pl (K.B.); mjamka@ump.edu.pl (M.J.); mduszuchowska@ump.edu.pl (M.D.-Ż.); karl-heinz.herzig@oulu.fi (K.-H.H.); 2 Department of Organization and Management in Health Care, Poznan University of Medical Sciences, Przybyszewskiego Str. 39, 60-356 Poznań, Poland; dariuszwalkowiak@ump.edu.pl; 3 Research Unit of Biomedicine and Internal Medicine, Biocenter of Oulu, Medical Research Center, Oulu University Hospital, University of Oulu, Aapistie Str. 5, 90220 Oulu, Finland

**Keywords:** phenylketonuria, PKU, vitamin status, vitamins, nutrition, metabolism, diet therapy, meta-analysis

## Abstract

The published data on the vitamin status of patients with phenylketonuria (PKU) is contradictory; therefore, this systematic review and meta-analysis evaluated the vitamin status of PKU patients. A comprehensive search of multiple databases (PubMed, Web of Sciences, Cochrane, and Scopus) was finished in March 2024. The included studies compared vitamin levels between individuals diagnosed with early-treated PKU and healthy controls while excluding pregnant and lactating women, untreated PKU or hyperphenylalaninemia cases, control groups receiving vitamin supplementation, PKU patients receiving tetrahydrobiopterin or pegvaliase, and conference abstracts. The risk of bias in the included studies was assessed by the Newcastle–Ottawa scale. The effect sizes were expressed as standardised mean differences. The calculation of effect sizes with 95% CI using fixed-effects models and random-effects models was performed. A *p*-value < 0.05 was considered statistically significant. The study protocol was registered in the PROSPERO database (CRD42024519589). Out of the initially identified 11,086 articles, 24 met the criteria. The total number of participants comprised 770 individuals with PKU and 2387 healthy controls. The meta-analyses of cross-sectional and case–control studies were conducted for vitamin B12, D, A, E, B6 and folate levels. PKU patients demonstrated significantly higher folate levels (random-effects model, SMD: 1.378, 95% CI: 0.436, 2.320, *p* = 0.004) and 1,25-dihydroxyvitamin D concentrations (random-effects model, SMD: 2.059, 95% CI: 0.250, 3.868, *p* = 0.026) compared to the controls. There were no significant differences in vitamin A, E, B6, B12 or 25-dihydroxyvitamin D levels. The main limitations of the evidence include a limited number of studies and their heterogeneity and variability in patients’ compliance. Our findings suggest that individuals with PKU under nutritional guidance can achieve a vitamin status comparable to that of healthy subjects. Our study provides valuable insights into the nutritional status of PKU patients, but further research is required to confirm these findings and explore additional factors influencing vitamin status in PKU.

## 1. Introduction

Phenylketonuria (PKU) is an inborn error of amino acid metabolism caused by mutations in the phenylalanine hydroxylase gene (PAH) [1]. It has a global prevalence of 1 in 23,930 live births, ranging from 1 in 4500 (Italy) to 1 in 125,000 (Japan) [2]. Normally, PAH catalyses the conversion of phenylalanine to tyrosine by the para-hydroxylation of the aromatic side chain [3], but the deficiency or absence of the liver enzyme PAH impairs this hydroxylation. Blood phenylalanine accumulates in toxic concentrations in the brain and leads to irreversible intellectual disabilities, behavioural abnormalities, motor dysfunctions and other health issues [4]. In 1953, Bickel et al. [5] first reported the efficacy of a diet low in phenylalanine in a child diagnosed with PKU, making it the first metabolic disorder that could be treated by a special diet. Since neurological damage occurs early after birth, it is strongly advocated to start treatment at up to 10 days of age [6]. Two milestones in PKU treatment are the early identification of the condition and the ongoing commitment to a carefully controlled dietary regimen throughout the patient’s life. The main goal is to strike a balance that ensures adequate nutrition while preventing the toxic accumulation of phenylalanine. This is achieved through the strict control of natural protein/phenylalanine intake, supplying essential amino acids by substituting natural proteins with phenylalanine-free amino acid mixtures and attaining normal growth and nutritional status [7]. Systematic follow-up, the close monitoring of phenylalanine intake, regular blood tests, and adjustments to the diet or medical formula are vital aspects of this treatment [8].

PKU patients must be supplemented with medical food substitutes containing the correct mix of essential amino acids, vitamins, minerals, and trace nutrients that would ordinarily be consumed in restricted foods [1]. However, similar to other chronic diseases, treatment adherence tends to decline over time [9,10]. Despite the broader availability of various medical foods, specialised low-protein foods, and approved medications, adhering to recommended phenylalanine goals continues to be a challenge, particularly for adolescents and older patients [9,11,12,13]. This could stem from a lack of understanding regarding dietary restrictions, low motivation to comply with treatment, challenges in coping with the disease [14], organoleptic properties of Phe-free food, the cost of formulated end products [15], psychosocial and emotional factors, family cohesion issues, parental commitment to sustaining the diet, understanding the disease, attitudes to healthcare professionals, and a lack of reimbursement of special dietary foods (in some healthcare systems) [4,16].

PKU patients may be at risk of developing deficiencies in various nutrients, including vitamins D [17,18], B12 [19,20,21,22], K [23], as well as zinc and selenium [22,24]. Vitamins play pivotal roles in various physiological processes, and any disturbances in their levels may have significant implications for overall health. Therefore, it is crucial to assess the impact of the PKU dietary regimen on vitamin status in these individuals. However, studies examining the vitamin status of PKU patients have reported conflicting findings. Some studies found elevated vitamin levels in PKU patients [17,25,26], while others have reported lower levels [27,28,29]. Additionally, certain studies found no significant differences in vitamin levels between PKU patients and healthy controls [30,31,32]. Given these contradictory results, this systematic review aims to comprehensively assess the vitamin status in patients with PKU compared to healthy individuals. We hypothesised that patients with PKU exhibited no differences in vitamin levels compared to healthy controls.

## 2. Methods

This systematic review and meta-analysis evaluated the vitamin status of patients with PKU compared to healthy controls.

### 2.1. Protocol and Registration

This study presentation followed the Preferred Reporting Items for Systematic Reviews and Meta-Analyses (PRISMA) [33] and the Cochrane [34] guidelines. It is registered with the International Prospective Register of Systematic Reviews (PROSPERO) under the registration number CRD42024519589 [35].

### 2.2. Inclusion and Exclusion Criteria

The search strategy was limited to human studies published in English in a peer-reviewed journal. Eligibility criteria for inclusion in the meta-analysis comprised both observational and intervention-based studies. These studies had to specifically compare vitamin levels between individuals diagnosed with PKU in the neonatal period, necessitating a phenylalanine-restricted diet and early treatment, and healthy individuals. To be considered, studies had to provide extractable data of interest, including an identified number of participants, their characteristics, and vitamin data for both groups.

The exclusion criteria were as follows: studies involving pregnant and lactating women; investigations focused on untreated PKU; hyperphenylalaninemia; studies where the control group was supplemented with vitamins under review; PKU patients receiving tetrahydrobiopterin (BH4) or pegvaliase; and conference publications and abstract-only papers.

### 2.3. Data Collection Process, Extraction and Analysis

The references were managed using Zotero, which is an open-source reference management software (Zotero, version 6.0.30, https://www.zotero.org/, accessed on 10 February 2024). The data selection process involved independent reviewers (K.B. and M.J.) who assessed publications based on predefined exclusion and inclusion criteria and occurred over three stages: the initial screening of the title, followed by examination of the abstract, and, finally, a comprehensive review of the full text. Screening titles and abstracts of the retrieved papers led to the exclusion of duplicates and clearly ineligible studies. Any study deemed valuable by at least one of the analysts proceeded to the next stage. In instances of uncertainties or disagreements during the selection process, a consensus was reached within the review team [33].

Each publication underwent a rigorous critical analysis, and the authors were contacted for clarification if any articles lacked information on the time of PKU diagnosis. Meta-analyses were performed to examine the evidence concerning the vitamin status of individuals with PKU and to assess the variations compared to the healthy population. This systematic review presented results for vitamins that were assessed in at least two identified studies. Quantitative analysis was performed in the form of meta-analysis.

### 2.4. Data Item

The following information was extracted from each included article:

1. General information: the title of the article, journal name, main author, and publication year.
2. Study characteristics: the study name and design, country (region), and sample size (total number of subjects and the number in each group who were included and completed the study).
3. Study population characteristics: age, sex, body mass index (BMI, kg/m^2^).
4. Description of dietary treatment: natural protein intake (g/day), protein substitute intake (g/day), total protein intake (g/day), phenylalanine intake (mg/d), annual mean/median phenylalanine levels (μmol/L), follow-up (yes or no), treatment adherence (yes or no), phenylalanine levels (μmol/L), tyrosine levels (μmol/L), and total protein levels (g/dL).
5. Main outcomes: blood or plasma levels of folate (nmol/L), folic acid (nmol/L), erythrocyte folate (nmol/L), total folate (nmol/L), vitamin B12 (pmol/L), 25-hydroxyvitamin D (nmol/L), 25-hydroxyvitamin D3 (nmol/L), cholecalciferol (nmol/L), 1,25-hydroxyvitamin D (nmol/L), vitamin A (μmol/L), beta-carotene (μmol/L), vitamin E (μmol/L), erythrocyte tocopherol (μmol/L), vitamin B6 (μmol/L), vitamin K (μmol/L), vitamin C (μmol/L), and biotin (ng/L).

If the levels of a vitamin were not analysed in any of the included papers, it was not considered in this study.

### 2.5. Information Sources and Search Strategy

The databases PubMed (Medline), Scopus, Web of Science, and the Cochrane Library were systematically searched for studies providing data on vitamin status (blood levels) in PKU patients compared to healthy controls in March 2024. The analysis encompassed experimental (randomised or non-randomised controlled trials) and observational (case–control and cross-sectional studies) studies without restrictions on publication year. References from previous review articles and the reference lists of the relevant papers were examined to identify any studies that might have been overlooked and any relevant articles that might not have been captured by the initial database search. 

In developing the search strategy, we employed a rigorous approach to identify relevant keywords, synonyms, and subject-indexing terms. Our process began with a review of the existing literature related to the topic of interest. Through this initial exploration, we identified key terms commonly used in the field, such as “PKU”, “phenylketonuria”, “vitamin”, “diet”, and “nutrition”. Additionally, we reviewed previously published systematic reviews and meta-analyses in the field to identify additional relevant terms and concepts. The terms in the titles and keywords of published articles were screened. We also expanded our scope to include synonyms and alternative terminology associated with PKU and vitamins. This involved consulting medical subject heading (MeSH) terms and databases of synonyms. The index terms were as follows:

Cochrane: “phenylketonuria” OR “phenylalanine hydroxylase deficiency” OR “phenylalanine hydroxylase deficient” OR “PKU” OR “hyperphenylalaninaemia” OR “BH4 deficiency” OR “BH4 deficient” OR “tetrahydrobiopterin deficiency” OR “tetrahydrobiopterin deficient” OR “PAH deficiency” OR “PAH deficient” OR “phenylketonuric” OR “hyperphenylalaninaemic” in Title Abstract Keyword AND “dietary” OR “supplement” OR “supplementations” OR “supplementation” OR “nutritional” OR “nutrition” OR “diet” OR “diets” OR “vitamin” OR “vitamins” OR “vitaminization” OR “vitaminisation” OR “nutrient” OR “nutrients” OR “micronutrient” OR “micronutrients” OR “fat-soluble” OR “fat soluble” OR “water soluble” OR “water-soluble” OR “calciferol” OR “cholecalciferol” OR “colecalciferol” OR “ergocalciferol” OR “dihydroxycholecalciferol” OR “1,25(OH)2D” OR “hydroxyvitamin” OR “25(OH)D” OR “hydroxyergocalciferol” OR “tocopherol” OR “tocopherols” OR “antioxidant” OR “antioxidants” OR “carotenoid” OR “carotenoids” OR “retinol” OR “retinal” OR “retinoic” OR “retinyl” OR “carotene” OR “provitamin” OR “provitamins” OR “phytomenadione” OR “phytonadione” OR “menaquinone” OR “menadione” OR “thiamine” OR “thiamin” OR “aneurin” OR “riboflavin” OR “niacinamide” OR “niacin” OR “nicotinic” OR “nicotinamide” OR “pantothenic” OR “pyridoxine” OR “pyridoxal” OR “pyridoxamine” OR “biotin” OR “folic” OR “folate” OR “folacin” OR “pteroylglutamic” OR “Pteroyl-L-glutamate” OR “Pteroyl-L-glutamic” OR “cobalamin” OR “cobalamins” OR “cobalamine” OR “cobalamines” OR “cyanocobalamin” OR “cyanocobalamine” OR “ascorbic” OR “ascorbate” OR “choline” in the title, abstract and keywords sections—(March 2024).

PubMed: (“phenylketonuria” OR “phenylalanine hydroxylase deficiency” OR “phenylalanine hydroxylase deficient” OR “PKU” OR “hyperphenylalaninaemia” OR “BH4 deficiency” OR “BH4 deficient” OR “tetrahydrobiopterin deficiency” OR “tetrahydrobiopterin deficient” OR “PAH deficiency” OR “PAH deficient” OR “phenylketonuric” OR “hyperphenylalaninaemic” [MeSH Terms]) AND (“dietary” OR “supplement” OR “supplementations” OR “supplementation” OR “nutritional” OR “nutrition” OR “diet” OR “diets” OR “vitamin” OR “vitamins” OR “vitaminization” OR “vitaminisation” OR “nutrient” OR “nutrients” OR “micronutrient” OR “micronutrients” OR “fat-soluble” OR “fat soluble” OR “water soluble” OR “water-soluble” OR “calciferol” OR “cholecalciferol” OR “colecalciferol” OR “ergocalciferol” OR “dihydroxycholecalciferol” OR “1,25(OH)2D” OR “hydroxyvitamin” OR “25(OH)D” OR “hydroxyergocalciferol” OR “tocopherol” OR “tocopherols” OR “antioxidant” OR “antioxidants” OR “carotenoid” OR “carotenoids” OR “retinol” OR “retinal” OR “retinoic” OR “retinyl” OR “carotene” OR “provitamin” OR “provitamins” OR “phytomenadione” OR “phytonadione” OR “menaquinone” OR “menadione” OR “thiamine” OR “thiamin” OR “aneurin” OR “riboflavin” OR “niacinamide” OR “niacin” OR “nicotinic” OR “nicotinamide” OR “pantothenic” OR “pyridoxine” OR “pyridoxal” OR “pyridoxamine” OR “biotin” OR “folic” OR “folate” OR “folacin” OR “pteroylglutamic” OR “pteroyl-L-glutamate” OR “Pteroyl-L-glutamic” OR “cobalamin” OR “cobalamins” OR “cobalamine” OR “cobalamines” OR “cyanocobalamin” OR “cyanocobalamine” OR “ascorbic” OR “ascorbate” OR “choline” [MeSH Terms])—(March 2024).

Scopus: (TITLE-ABS-KEY (“phenylketonuria” OR “phenylalanine hydroxylase deficiency” OR “phenylalanine hydroxylase deficient” OR “PKU” OR “hyperphenylalaninaemia” OR “BH4 deficiency” OR “BH4 deficient” OR “tetrahydrobiopterin deficiency” OR “tetrahydrobiopterin deficient” OR “PAH deficiency” OR “PAH deficient” OR “phenylketonuric” OR “hyperphenylalaninaemic”) AND TITLE-ABS-KEY (“dietary” OR “supplement” OR “supplementations” OR “supplementation” OR “nutritional” OR “nutrition” OR “diet” OR “diets” OR “vitamin” OR “vitamins” OR “vitaminization” OR “vitaminisation” OR “nutrient” OR “nutrients” OR “micronutrient” OR “micronutrients” OR “fat-soluble” OR “fat soluble” OR “water soluble” OR “water-soluble” OR “calciferol” OR “cholecalciferol” OR “colecalciferol” OR “ergocalciferol” OR “dihydroxycholecalciferol” OR “1,25(OH)2D” OR “hydroxyvitamin” OR “25(OH)D” OR “hydroxyergocalciferol” OR “tocopherol” OR “tocopherols” OR “antioxidant” OR “antioxidants” OR “carotenoid” OR “carotenoids” OR “retinol” OR “retinal” OR “retinoic” OR “retinyl” OR “carotene” OR “provitamin” OR “provitamins” OR “phytomenadione” OR “phytonadione” OR “menaquinone” OR “menadione” OR “thiamine” OR “thiamin” OR “aneurin” OR “riboflavin” OR “niacinamide” OR “niacin” OR “nicotinic” OR “nicotinamide” OR “pantothenic” OR “pyridoxine” OR “pyridoxal” OR “pyridoxamine” OR “biotin” OR “folic” OR “folate” OR “folacin” OR “pteroylglutamic” OR “pteroyl-l-glutamate” OR “pteroyl-l-glutamic” OR “cobalamin” OR “cobalamins” OR “cobalamine” OR “cobalamines” OR “cyanocobalamin” OR “cyanocobalamine” OR “ascorbic” OR “ascorbate” OR “choline”)—(March 2024).

Web of Science: “phenylketonuria” OR “phenylalanine hydroxylase deficiency” OR “phenylalanine hydroxylase deficient” OR “PKU” OR “hyperphenylalaninemia” OR “BH4 deficiency” OR “BH4 deficient” OR “tetrahydrobiopterin deficiency” OR “tetrahydrobiopterin deficient” OR “PAH deficiency” OR “PAH deficient” OR “phenylketonuric” OR “hyperphenylalaninaemia” (Topic) AND “dietary” OR “supplement” OR “supplementations” OR “supplementation” OR “nutritional” OR “nutrition” OR “diet” OR “diets” OR “vitamin” OR “vitamins” OR “vitaminization” OR “vitaminisation” OR “nutrient” OR “nutrients” OR “micronutrient” OR “micronutrients” OR “fat-soluble” OR “fat soluble” OR “water soluble” OR “water-soluble” OR “calciferol” OR “cholecalciferol” OR “colecalciferol” OR “ergocalciferol” OR “dihydroxycholecalciferol” OR “1,25(OH)2D” OR “hydroxyvitamin” OR “25(OH)D” OR “hydroxyergocalciferol” OR “tocopherol” OR “tocopherols” OR “antioxidant” OR “antioxidants” OR “carotenoid” OR “carotenoids” OR “retinol” OR “retinal” OR “retinoic” OR “retinyl” OR “carotene” OR “provitamin” OR “provitamins” OR “phytomenadione” OR “phytonadione” OR “menaquinone” OR “menadione” OR “thiamine” OR “thiamin” OR “aneurin” OR “riboflavin” OR “niacinamide” OR “niacin” OR “nicotinic” OR “nicotinamide” OR “pantothenic” OR “pyridoxine” OR “pyridoxal” OR “pyridoxamine” OR “biotin” OR “folic” OR “folate” OR “folacin” OR “pteroylglutamic” OR “pteroyl-L-glutamate” OR “Pteroyl-L-glutamic” OR “cobalamin” OR “cobalamins” OR “cobalamine” OR “cobalamines” OR “cyanocobalamin” OR “cyanocobalamine” OR “ascorbic” OR “ascorbate” OR “choline” (Topic)—(March 2024).

### 2.6. Risk of Bias of Individual Studies

The risk of bias was evaluated using the Newcastle–Ottawa scale (NOS) [36], which is a tool for assessing the quality of non-randomised studies in meta-analyses, and the NOS adapted by Modesti et al. [37] was used for cross-sectional studies. The NOS assessment of case–control studies examined three key domains: the selection of study groups, the comparability of these groups, and the ascertainment of the exposure of interest. The modified NOS for cross-sectional studies included an assessment of the following three perspectives: potential bias on selection, comparability, and outcome. Each study underwent an independent assessment by two reviewers (K.B. & M.J.) to ensure consistency in the application of the tool. The reviewers evaluated the risk of bias for each study based on the predefined criteria within the NOS, and any discrepancies were addressed through discussion to reach a consensus. The NOS assigns scores to each study, indicating the overall methodological quality. For cross-sectional studies, the overall score ranged from 0 to 10, while for case–control studies, it ranged from 0 to 9. A study with a score of ≥7 was considered to possess a low risk of bias, scores ranging from 5 to 6 were considered a moderate risk of bias, and scores ≤ 4 were a high risk of bias.

### 2.7. Statistical Analysis

The meta-analysis was conducted using Comprehensive Meta-Analysis Software, version 3.0 (Biostat, Inc., Englewood, CO, USA) to compare vitamin status in PKU patients vs. healthy control individuals if at least two identified studies per vitamin were available. If several studies incorporated data from the same population on the same outcome, only one paper was included in the meta-analysis. Vitamin plasma and serum levels, erythrocyte folate, and tocopherol levels were used for the effect size measure. A *p*-value < 0.05 was considered statistically significant. The metrics for effect size computation were the mean and standard deviations (SD), and for studies reporting the standard error of the mean (SEM), the SD was derived by multiplying the reported SEM by the square root of the corresponding sample size. When a 95% confidence interval (CI) was available, the SD for each group was calculated by dividing the CI by 3.92 and multiplying the result by the square root of the sample size for each group. If the data were available as a median and interquartile range, the approximate SD was calculated by dividing the width of the interquartile range by 1.35. When studies included multiple intervention groups, they were combined into one group following the Cochrane guidelines. If the data were presented as a median and range, and additional data were not obtained, the study was excluded from the meta-analysis. The meta-analysis was conducted using the original data presented in the included studies. Heterogeneity among studies was assessed using the Cochran Q statistic (*p* < 0.1 indicates significant heterogeneity) and the I^2^ test (<25%, 25–75%, and >75% denoted low, moderate, and high heterogeneity, respectively). The data and calculated effect sizes were combined with 95% CI using fixed-effect models when no heterogeneity was observed. For outcomes with moderate to high heterogeneity, random-effects models were employed. The effect sizes were expressed as standardised mean differences (SMDs) to facilitate comparisons across studies. SMDs were determined by dividing the difference between the mean outcome values of groups by the pooled SD of the outcome values. Forest plots were generated to visualise the study-specific effect sizes and 95% CI. Sensitivity analyses were conducted by systematically removing each study one at a time and recalculating the pooled estimates to assess the robustness of the findings. Additionally, studies with a high risk of bias were excluded from the analysis to check the effect of bias on the results and assess its potential influence on the overall findings. Funnel plots were generated, and Begg’s and Egger’s tests were employed to assess the presence of publication bias. A cumulative meta-analysis was conducted along with subgroup analyses. Subgroups were defined based on the mean age (<18 years vs. ≥18 years), development status of countries (developed vs. developing), and study year (≤2000 vs. >2000). PKU individuals have specific dietary regimens and different growth rates and nutritional requirements during childhood compared to adulthood, which may affect their vitamin levels. The comparison of older and more recent data was applied to explore potential trends or shifts in vitamin levels among PKU patients over time. This aligns with the historical context of PKU management, considering that significant advancements in treatment and dietary guidelines have occurred over time. Subgroup analyses based on the development status of countries were performed to elucidate potential variations in the vitamin status of PKU patients across different healthcare settings and economic standards.

## 3. Results

### 3.1. Search Results

**Figure 1:**
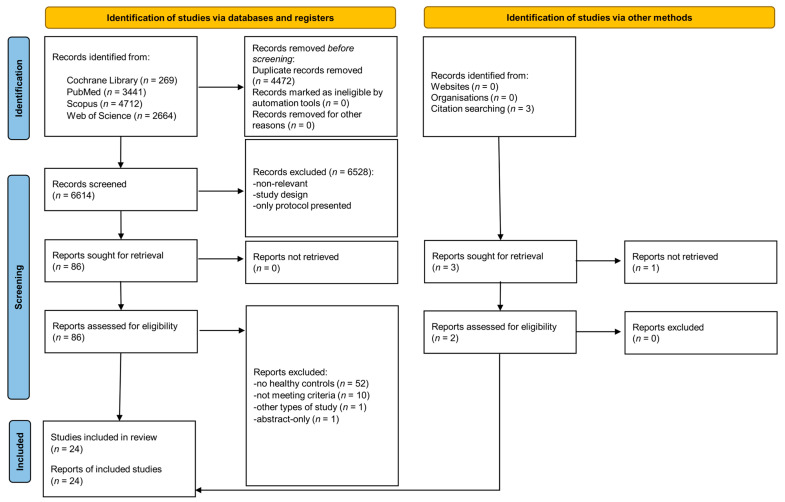
PRISMA 2020 flow diagram.

Out of the initially identified 11,086 articles, 4472 duplicates were excluded, and the screening of titles and abstracts identified 88 relevant articles of which 24 met the inclusion criteria (Figure 1).

### 3.2. Study Characteristics

The included studies were published from 1994 [38] to 2023 [39], and their characteristics are detailed in Table 1. Four studies were conducted in Greece [25,27,40,41], three in Turkey [31,42,43], three in Spain [32,44,45], two in Austria [26,46], two in Chile [39,47], two in Japan [29,30], two in the United Kingdom [48,49], two in the USA [38,50], and one each in Germany [51], France [52], Poland [28], and Switzerland [53]. All studies, except for one case–control study [26], were cross-sectional and compared individuals with PKU to healthy controls. The PKU groups varied from 10 [39,52] to 107 [28], while control groups ranged from 6 [38] to 1676 [49]. Five studies assessed children [25,27,28,31,40], one studied adolescents [43], children and adolescents were included in nine studies [26,32,38,41,42,45,46,50,51,53], six studies examined adults [29,30,39,48,49,52], and the remaining two studies had a mixed population [44,47]. Most studies included males and females [24,26,27,28,29,30,37,38,40,41,42,45,46,49,50,51,52], and while it was not explicitly stated in other studies [25,27,40,44,45,48,49], they likely involved both male and female participants. Table 2 presents the characteristics of the diet and metabolic status of the studied individuals.

### 3.3. Comparison of Folate Levels

**Figure 2:**
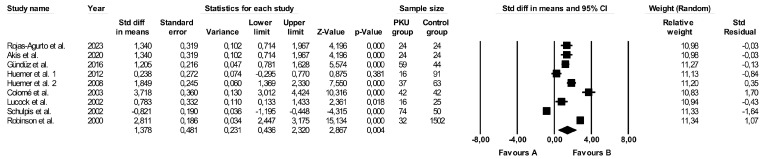
Forest plot of the folate levels in PKU patients (favours A) vs. controls (favours B) (random model) [26,27,39,42,43,44,46,48,49]. CI—confidence interval; Std—standard; Std diff—standard differences.

Folate levels were evaluated in ten studies [26,27,39,42,43,44,46,48,49,51] (Table 3), nine of which [26,27,39,42,43,44,46,48,49] were incorporated into the meta-analysis and one study [51] was excluded because the data were presented as the median and range. The results showed significantly higher folate concentrations in PKU patients compared to healthy controls (random-effects model, SMD: 1.378, 95% CI: 0.436, 2.320, *p* = 0.004, Figure 2). The assessment of heterogeneity indicated a high risk (Q-value = 257.997, *p* < 0.001, I^2^ = 96.899%). Figure S1 displays a funnel plot of the standard error with standard differences in the means of folate levels. Figure S2 shows the outcomes of the sensitivity analysis, while Figure S3 illustrates the cumulative analysis results. After removing studies with a high risk of bias, the discrepancy in folate levels in PKU and the controls persisted, demonstrating a statistically significant difference (random-effects model, SMD: 1.423, 95% CI: 0.232, 2.614, *p* = 0.019, Figure S4).

### 3.4. Comparison of Vitamin B12 Levels

**Figure 3:**
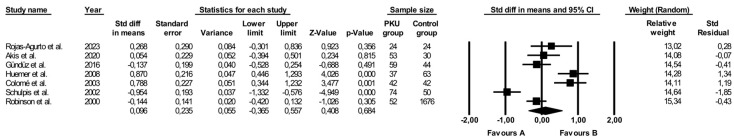
Forest plot of vitamin B12 levels in PKU patients (favours A) vs. controls (favours B) (random model) [26,27,39,42,43,44,49]. CI—confidence interval; Std—standard; Std diff—standard differences.

The comparison of vitamin B12 levels in PKU patients and healthy individuals in analysis did not incorporate data from one study [51] due to their presentation of median and range data, leaving seven studies [26,27,39,42,43,44,49] and revealing no significant difference between the PKU and control groups (random-effects model, SMD: 0.096, 95% CI: −0.365, 0.557, *p* = 0.684, Figure 3). The risk of heterogeneity among the studies was high (Q-value = 70.821, *p* < 0.001, I^2^ = 91.528%). A funnel plot of the standard error by standard differences in the mean vitamin B12 levels is shown in Figure S5. Figure S6 illustrates the findings from the sensitivity analysis, while Figure S7 displays the results of the cumulative analysis. The exclusion of studies with a high risk of bias did not change the results (random-effects model, SMD: 0.142, 95% CI: −0.439, 0.722, *p* = 0.632, Figure S8).

### 3.5. Comparison of Vitamin D Levels

**Figure 4:**
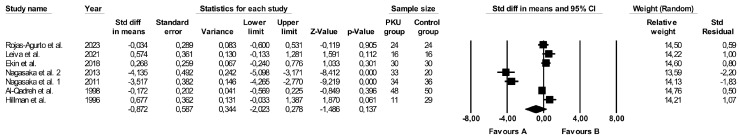
Forest plot of vitamin D levels in PKU patients (favours A) vs. controls (favours B) (random model) [29,30,31,39,41,47,50]. CI—confidence interval; Std—standard; Std diff—standard differences.

The vitamin D levels among PKU patients and healthy controls were evaluated in seven articles [29,30,31,39,41,47,50] by pooling data from studies that provided information on 25-hydroxyvitamin D [30,39,41,47,50], 25-hydroxyvitamin D3 [29], and cholecalciferol [31]. The meta-analysis showed no significant differences in vitamin D levels between the PKU and control groups (random-effects model, SMD: −0.872, 95% CI: −2.023, 0.278, *p* = 0.137, Figure 4). There was high heterogeneity between the studies (Q-value = 150.122, *p* < 0.001, I^2^ = 96.003%). A funnel plot of the standard errors by standard differences for the mean vitamin D levels is shown in Figure S9. The results of the sensitivity analysis are shown in Figure S10, and the results of cumulative analysis are presented in Figure S11. The exclusion of studies considered to have a high risk of bias did not alter the findings (random-effects model, SMD: −1.343, 95% CI: −3.073, 0.386, *p* = 0.128, Figure S12).

### 3.6. Comparison of 1,25-dihydroxyvitamin D Levels

**Figure 5:**
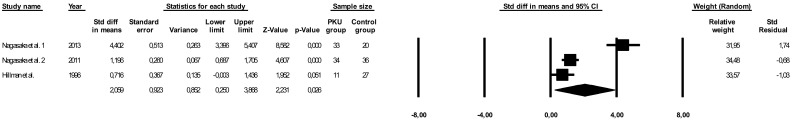
Forest plot of 1,25-dihydroxyvitamin D levels in PKU patients (favours A) vs. controls (favours B) (random model) [29,30,50]. CI—confidence interval; Std—standard; Std diff—standard differences.

The meta-analysis of the three studies [29,30,50], which evaluated 1,25-dihydroxyvitamin D concentrations, revealed significantly higher levels in the PKU group compared to the controls (random-effects model, SMD: 2.059, 95% CI: 0.250, 3.868, *p* = 0.026, Figure 5). The risk of heterogeneity was assessed as high (Q-value = 37.916, *p* < 0.001, I^2^ = 94.725%). A funnel plot of the standard error by standard differences in mean 1,25-dihydroxyvitamin D concentrations is shown in Figure S13. Figure S14 shows the outcomes of the sensitivity analysis, while Figure S15 presents the cumulative analysis results. Excluding the study with a high risk of bias revealed no significant differences between the groups (random-effects model, SMD: 2.768, 95% CI: −0.373, 5.909, *p* = 0.084, Figure S16).

### 3.7. Comparison of Vitamin A Levels

**Figure 6:**
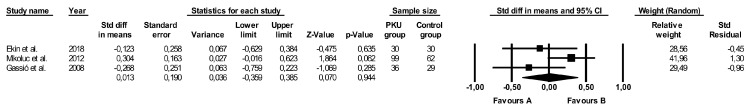
Forest plot of vitamin A levels in PKU patients (favours A) vs. controls (favours B) (random model) [28,31,32]. CI—confidence interval; Std—standard; Std diff—standard differences.

Vitamin A levels were assessed in four studies [28,31,32,52], but one study was excluded [52] because the data were presented as the median and range. There were no significant differences in vitamin A concentrations between the PKU and healthy individuals (random-effects model, SMD: 0.013, 95% CI: −0.359, 0.385, *p* = 0.944, Figure 6). The risk of heterogeneity was assessed as moderate (Q-value = 4.427, *p* = 0.109, I2 = 54.824%). A funnel plot of the standard error by standard differences for the means of vitamin A concentrations is shown in Figure S17. Figure S18 illustrates the findings from the sensitivity analysis, while Figure S19 displays the results of the cumulative analysis. After excluding studies with a high risk of bias, the results remained unchanged, with no significant differences between the groups (random-effects model, SMD: −0.198, 95% CI: −0.550, 0.155, *p* = 0.272, Figure S20).

### 3.8. Comparison of Vitamin E Levels

**Figure 7:**
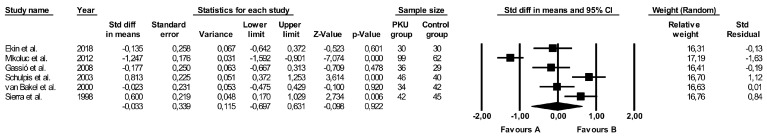
Forest plot of the vitamin E levels in PKU patients (favours A) vs. controls (favours B) (random model) [25,28,31,32,45,53]. CI—confidence interval; Std—standard; Std diff—standard differences.

Seven studies [25,28,31,32,45,52,53] compared the vitamin E concentrations of PKU and healthy individuals, but one paper [52] was not analysed due to the data presented as the median and range. The meta-analysis did not find significant differences (random-effects model, SMD: −0.033, 95% CI: −0.697, 0.631, *p* = 0.922, Figure 7), and the risk of heterogeneity was high (Q-value = 69.032, *p* < 0.001, I^2^ = 92.757%). A funnel plot of the standard error by standard differences in the means of vitamin E levels is shown in Figure S21. The results of sensitivity analysis are shown in Figure S22, and the results of cumulative analysis are presented in Figure S23. After excluding studies with a high risk of bias, there were no significant differences between the groups (random-effects model, SMD: 0.176, 95% CI: −0.483, 0.834, *p* = 0.601, Figure S24).

### 3.9. Comparison of Vitamin B6 Levels

**Figure 8:**
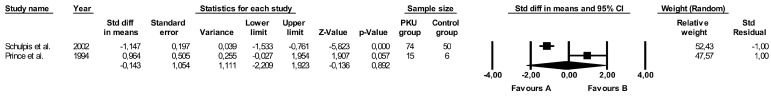
Forest plot of vitamin B6 levels in PKU patients (favours A) vs. controls (favours B) (random model) [27,38]. CI—confidence interval; Std—standard; Std diff—standard differences.

Only two studies compared vitamin B6 concentrations in PKU and healthy controls [27,38], and the meta-analysis showed no significant difference between the groups (random-effects model, SMD: −0.143, 95% CI: −2.209, 1.923, *p* = 0.892, Figure 8). The assessment of heterogeneity indicated a high risk (Q-value = 15.143, *p* < 0.001, I^2^ = 93.397%). Figure S25 presents the outcomes of the sensitivity analysis, while Figure S26 illustrates the cumulative analysis results.

### 3.10. Comparison of Other Vitamins

Other vitamin levels are presented in Table 3, but a meta-analysis was not feasible due to limited data availability as only one study measured beta-carotene levels [25], one study investigated vitamin K concentrations [31], and another assessed biotin [40]. In addition, one [52] of the two studies [25,52] that investigated vitamin C presented data as the median and range, precluding the possibility of conducting a meta-analysis. No studies assessing the blood levels of vitamin B1, vitamin B2, vitamin B3, vitamin B5, vitamin B7, and choline were identified.

### 3.11. Subgroup Analysis

The subgroup analysis results are presented in Figures S27–S40, showing a significant difference in folate levels between the PKU individuals and controls based on age (under the age of 18 vs. aged 18 years and above; random-effects model, SMD: 1.239, 95% CI: 0.063, 2.414, *p* = 0.039 vs. SMD: 1.666, 95% CI: 0.346, 2.986, *p* = 0.013, Figure S27). Upon stratifying the studies by developed and developing countries, it was revealed that folate levels were elevated in PKU patients compared to controls in both developing and developed countries (random-effects model, SMD: 1.270, 95% CI: 0.964, 1.576, *p* < 0.001 vs. SMD: 1.423, 95% CI: 0.004, 2.842, *p* = 0.049, Figure S28). The year of the study did not impact the folate levels as they were consistently higher in PKU patients regardless of whether the study was conducted in 2000 or before or after this date (random-effects model, SMD: 2.811, 95% CI: 2.447, 3.174, *p* < 0.001 vs. SMD: 1.192, 95% CI: 0.279, 2.106, *p* = 0.011, Figure S29). However, only one study was conducted before 2000 [49].

The age of participants did not appear to influence vitamin B12 levels, as there were no significant differences observed between older individuals with PKU and their healthy counterparts, nor between younger individuals with PKU and the controls (random-effects model, SMD: −0.016, 95% CI: −0.390, 0.357, *p* = 0.931 vs. SMD: 0.119, 95% CI: −0.557, 0.795, *p* = 0.730, Figure S30). Stratifying studies based on the development status of countries did not alter the findings, as no significant differences in vitamin B12 concentrations were observed in either developed or developing countries (random-effects model, SMD: 0.132, 95% CI: −0.657, 0.920, *p* = 0.744 vs. SMD: 0.013, 95% CI: −0.248, 0.275, *p* = 0.920, Figure S31). Stratifying the studies by year of publication did not reveal any significant differences in vitamin B12 levels between participants in earlier studies [49] (≤2000) and those in later studies (>2000) (random-effects model, SMD: −0.144, 95% CI: −0.420, 0.132, *p* = 0.305 vs. SMD: 0.142, 95% CI: −0.439, 0.722, *p* = 0.632, Figure S32).

The age subgroup analysis of 25-dihydroxyvitamin D concentrations yielded consistent results with no significant differences in either younger or older populations (random-effects model, SMD: 0.200, 95% CI: −0.626, 1.025, *p* = 0.636 vs. SMD: −1.827, 95% CI: −3.910, 0.255, *p* = 0.085, Figure S33). Studies conducted in developing and developed countries revealed no significant difference in 25-dihydroxyvitamin D concentrations in PKU patients compared to the controls (random-effects model, SMD: 0.231, 95% CI: −0.102, 0.564, *p* = 0.174 vs. SMD: −1.764, 95% CI: −3.880, 0.352, *p* = 0.102, Figure S34). The subgroup analysis of 25-dihydroxyvitamin D concentrations stratified by the year of the studies (≤2000 vs. >2000) revealed no differences in either subgroup (random-effects model, SMD: 0.199, 95% CI: −0.626, 1.025, *p* = 0.636 vs. SMD: −1.343, 95% CI: −3.073, 0.386, *p* = 0.128, Figure S35).

Stratifying studies based on age showed no significant differences in levels of 1,25-dihydroxyvitamin D in either younger or older populations (random-effects model, SMD: 0.716, 95% CI: −0.003, 1.436, *p* = 0.051 vs. SMD: 2.768, 95% CI: −0.373, 5.909, *p* = 0.084, Figure S36). Similarly, stratifying by the year of the study revealed no significant differences in subgroups (≤2000 vs. >2000) (random-effects model, SMD: 0.716, 95% CI: −0.003, 1.436, *p* = 0.051 vs. SMD: 2.768, 95% CI: −0.373, 5.909, *p* = 0.084, Figure S37). Subgroup analysis based on the development status of countries was not possible due to a lack of relevant studies.

Subgroup analysis based on the development status of countries revealed no significant differences in vitamin A levels between the PKU patients and controls both in developed and developing subgroups (random-effects model, SMD: 0.050, 95% CI: −0.507, 0.606, *p* = 0.862 vs. SMD: −0.123, 95% CI: 0.384, −0.475, *p* = 0.635, Figure S38). Subgroup analysis for vitamin A levels based on the age of participants and study year was not possible due to a lack of relevant studies. The development status of countries (developed vs. developing) did not affect vitamin E levels, as indicated by subgroup analysis (random-effects model, SMD: −0.012, 95% CI: −0.805, 0.780, *p* = 0.976 vs. SMD: −0.135, 95% CI: −0.642, 0.372, *p* = 0.601, Figure S39). The year of the study did not affect the findings, with no significant differences in vitamin E levels between the PKU patients and healthy individuals irrespective of whether the study was conducted before or after 2000 (random-effects model, SMD: 0.292, 95% CI: −0.318, 0.903, *p* = 0.348 vs. SMD: −0.193, 95% CI: −1.122, 0.737, *p* = 0.684, Figure S40). A subgroup analysis for vitamin A levels based on age was not possible due to a lack of relevant studies. The same limitation was encountered in subgroup analyses for vitamin B6 levels.

### 3.12. Relative Differences in Vitamin Levels across Studies

Table 4 displays the relative differences between the levels of each vitamin in PKU patients and healthy subjects for each study included. These relative values provide more clinical insight into the differences observed between the groups.

### 3.13. Risk of Bias

The risk of bias assessment is presented in Table 5, showing that the overall scores for cross-sectional studies ranged from high to moderate (0–6 points). The methodological and reported shortcomings observed in numerous studies are evident in the low scores, lowering the reliability of the studies reviewed. Regarding selection bias, most studies investigated selected population groups, with only 13% of studies considered as “somewhat representative of the average in the target population”. Most studies (87%) used validated measurement tools to assess vitamin levels, and three studies did not describe the chosen tools. Given that blood samples were obtained from all participants invited to take part in this study, indicating a complete response rate for blood sample collection, it can be inferred that there was no non-response among the participants approached for blood sample collection. Consequently, 82.6% of the studies received a score in this category. However, four studies either did not provide data or did not conduct tests for all patients. Regarding comparability, 70.8% of studies controlled at least one main confounding factor (age) and 62.5% applied additional factors, such as sex, BMI, nutritional status, weight, height, weight for height, relative weight, liver function test, Tanner stages, sex hormone, and the same geographical area. No studies provided data about whether the assessment of outcome was blinded, and only two studies (8.7%) included complete statistical tests, while the remaining studies (91.3%) did not present the CIs, resulting in a deduction in points in this category. The risk of bias for the case–control study [26] was assigned six points.

## 4. Discussion

Our meta-analysis indicates that individuals with PKU have significantly higher folate and 1,25-dihydroxyvitamin D concentrations compared to healthy controls, but there were no significant differences in 25-hydroxyvitamin D, vitamin A, E, B6, or B12 levels. These findings provide valuable insights into the overall vitamin status of PKU populations, suggesting that these vitamins may not be substantially affected by the treated PKU condition.

Previously, only one meta-analysis examined the status of nutrients crucial for brain development and functioning in individuals with PKU compared to the controls [54] using similar inclusion and exclusion criteria, but we searched only for PKU patients diagnosed at neonatal screening. They also reported no significant differences between groups in vitamin B12, E, and D levels. They presented forest plots on folate levels without the overall effect size due to excessively high heterogeneity within the data. They did not perform meta-analyses for vitamins A, C, and B6, as well as choline levels, due to their decision to perform them only if at least four studies were available for a specific nutrient, though they provided forest plots without the overall pooled effect. There are notable differences between our studies. Firstly, Montoya Parra et al. [54] published the results in 2018, and our meta-analysis included studies conducted later [31,39,42,47]. Secondly, we excluded some of their included studies because there was no information on neonatal diagnosis [55,56], and patients were diagnosed outside the neonatal programme in one study [57]. One paper [58] included by Montoya Parra et al. [54] was in Chinese, but we could not contact the authors. For choline analysis, Montoya Parra et al. [54] utilised studies [59,60] that reported brain choline concentrations, but one paper [60] presented data for the control group in the form of ranges; thus, a meta-analysis was not possible. Finally, Montoya Parra et al. [54] provided only forest plots and assessed heterogeneity, while our study also included sensitivity analyses, funnel plots, a publication bias and risk of bias assessment, cumulative meta-analyses, and subgroup analyses.

One of the main findings of our meta-analysis was the significantly higher folate concentrations observed in PKU patients, which may result from dietary education/practices among individuals with PKU. Despite the high heterogeneity among the studies, the difference in folate levels persisted even after sensitivity analysis, indicating the robustness of this finding. Subgroup analysis revealed insights into the folate status among PKU patients. Notably, regardless of age, individuals with PKU consistently exhibited higher folate levels compared to healthy controls. Our analysis also highlighted no geographic disparities, with significantly higher folate levels observed in PKU patients from both developing and developed countries compared to the controls. However, the *p*-value for the subgroup analysis conducted on developed countries was 0.049, suggesting that the geographical location may influence the magnitude of the difference in folate levels between PKU patients and healthy individuals, with the potential of slight variations between developed and developing regions. Rojas-Agurto et al. [39] compared adult PKU patients receiving continuous nutritional treatment with strict follow-up to those who discontinued their protein substitute intake at 18 years of age, reporting higher serum folic acid levels in both groups of PKU patients compared with controls. Similarly, Akis et al. [42] found higher folic acid levels in both the high-adherence and low-adherence groups compared to the control group (*p* < 0.001). Gündüz et al. [43] reported significantly higher folic acid concentrations in both well-controlled and poorly controlled PKU patients compared to the controls (*p* < 0.001) with no difference between PKU groups (*p* = 0.830). In the investigation of Huemer et al. [26], folate concentrations in PKU patients correlated significantly with their intake of amino acids, natural protein, total protein, and folic acid and were negatively correlated with Phe levels. PKU individuals were reported to have higher intakes of folic acid than the controls (*p* < 0.01) and were four times the upper reference and control group levels [39]. The high folate levels in PKU were associated with the substantial incorporation of vegetables in the Phe-restricted diet [20,26,49]. Vegetables, particularly dark green leafy varieties, along with fruits and fruit juices, are rich sources of folate [61]. The PKU diet primarily consists of vegetables and fruits, supplemented with low-protein dietetic products [62], often fortified with folic acid. Therefore, this outcome seems to align naturally.

Contrary to folate, there were no significant differences in vitamin B12 levels between PKU patients and healthy individuals. A comparison between continuously treated PKU patients and those who discontinued their protein substitute intake upon reaching the age of 18 years revealed significantly lower vitamin B12 levels in the latter group [39]. The second group of PKU patients maintained a predominantly vegan diet, which provided a limited amount of essential amino acids. Consequently, these PKU patients exhibited low blood vitamin B12 levels after discontinuing treatment, which could suggest that a formula-based diet is important for maintaining adequate levels of vitamin B12 [39]. Interestingly, Colomé et al. [44], when evaluating patients on amino acid mixtures, documented that those with poor dietary compliance had significantly lower vitamin B12 concentrations compared to those with good metabolic control. Robinson et al. [49] compared different PKU participants, revealing sufficient vitamin B12 levels in patients on a strict diet and significantly lower levels in participants on an unrestricted and relaxed diet compared to healthy individuals. These observations suggest that consuming supplemented PKU products results in better vitamin B12 status among PKU patients.

There were no significant differences in 25-dihydroxyvitamin D levels between the PKU patients and controls, and this was consistent across the subgroup analyses for age, the country’s development statuses, and study years. The concentrations of 1,25-dihydroxyvitamin D in PKU patients were initially observed to be significantly higher than in the controls, but no significant differences were found on the exclusion of a high risk of bias study. Therefore, vitamin D status may remain stable in PKU populations compared to healthy individuals despite demographic, age, or temporal variations. When comparing PKU patients taking a continuous nutritional treatment and strict follow-up to those who discontinued their protein substitute intake at 18 years of age, Rojas-Agurto et al. [39] found that serum vitamin D3 levels were significantly lower in the latter group compared to the former, implying that ongoing nutritional management and adherence to treatment protocols are sufficient to maintain adequate vitamin D levels in individuals with PKU. Interestingly, Nagasaka et al. [30] found 1,25-dihydroxyvitamin D levels to be significantly higher in adults diagnosed with PKU compared to the controls, while levels of 25-hydroxyvitamin D were significantly lower than the controls. According to the Endocrine Society Clinical Practice Guideline on vitamin D deficiency [63], serum levels of 1,25-dihydroxyvitamin D do not accurately reflect vitamin D reserves and are not considered useful for defining vitamin D status. Even in cases of vitamin D deficiency, serum 1,25-dihydroxyvitamin D levels may appear normal or elevated due to secondary hyperparathyroidism. Therefore, while its measurement is valuable in assessing acquired and inherited disorders related to the metabolism of 25-hydroxyvitamin D and phosphate, it does not provide a reliable indicator of overall vitamin D status; 25-hydroxyvitamin D should be measured instead. However, keeping in mind recent advances, vitamin D metabolism may be more complex [64]. Potential high levels of 1,25-dihydroxyvitamin D in PKU patients are very interesting and may form the basis for future investigations.

Our analysis also explored the status of other vitamins, including vitamins A, E, and B6, but observed no significant differences between PKU patients and controls. Subgroup analyses based on various factors did not alter the overall non-significant findings, suggesting that these nutrients may not be affected by the PKU condition or its treatment regimen.

This meta-analysis is one of the first to investigate the vitamin status of PKU patients compared to healthy individuals. Through the use of strict criteria and including only individuals diagnosed during the neonatal period, this present study ensures a more homogeneous and well-defined study population. This consistency in the timing of diagnosis and initiation of treatment helps to reduce variability among participants and ensures that they receive similar management strategies from infancy. Furthermore, this restriction helps to minimise confounding factors and ensures that the observed differences in outcomes between individuals with PKU and healthy controls are more likely attributable to the condition rather than variations in diagnostic timing. By stratifying the data based on age, the development status of countries, and study years, we aimed to uncover potential nuances that could impact the interpretation of our meta-analysis findings.

It is essential to address the concerns regarding the risk of bias identified during our assessments, particularly in domains such as selection bias and outcome ascertainment. Selection bias was primarily associated with the representativeness of the sample and its size. The evaluation of outcomes, as per NOS criteria, hinged on the assessment of the outcome and the statistical tests employed. Points in the first category were granted only if “independent blind assessment”, “record linkage”, or “self-report” methods were implemented in the study. Since the measurement of vitamin levels was objective, it should not impact the results; however, we were unable to grant any points in this category, resulting in lower overall scores for all studies. In the statistical category, the absence of points was primarily due to the lack of CIs. However, it is important to note that in other aspects, statistical analysis was fully described and adequate. Some limitations are associated with the assessment tool itself. The NOS relies on subjective judgment in assigning scores for each domain, potentially introducing variability in assessments between reviewers. Moreover, the original NOS primarily focuses on study design features and overlooks other potential sources of bias, such as selective outcome reporting or conflicts of interest, which could affect the validity of the study findings. While the NOS allows for the assessment of study quality, it lacks a quantitative measure of bias magnitude, making it difficult to determine the extent to which bias may have influenced the study outcomes. Finally, we conducted a publication bias assessment despite the limited number of studies included in the review. However, when the number of studies was smaller than 10, publication bias could not be reliably assessed. These limitations underscore the need for the cautious interpretation of our findings.

Various factors may influence our findings. Research in the PKU field, as well as in any rare disease, is particularly challenging, primarily due to the small patient population. The small number of individuals affected by PKU restricts the available pool of research participants. Only Leiva et al. [47] justified and calculated the required sample size. Differentiated compliance among PKU patients, as well as the severity of PKU, could impact study outcomes. Environmental factors, such as sunlight exposure and regional dietary patterns, may contribute to variations in vitamin status among the study participants. Furthermore, changes in treatment strategies or the composition of formulas could also influence the observed outcomes. Patient demographics, including age, sex, and ethnicity, varied across the included studies, potentially introducing heterogeneity in outcomes.

Meta-regression and network meta-analysis, as well as subgroup analysis regarding the sex, type of protein substitute, adherence to treatment, and metabolic control of participants, were not performed. Some subgroups contained only a small number of included studies, which could have influenced the results. Further larger studies are needed to validate our findings.

## 5. Conclusions

In conclusion, the present meta-analysis revealed that PKU patients have higher folate and 1,25-dihydroxyvitamin D levels compared to the controls. No differences in vitamin A, E, B6, B12, and 25-dihydroxyvitamin D levels between PKU patients and healthy subjects were documented. This suggests that individuals with PKU can attain adequate vitamin status similar to that of their healthy counterparts. Comparable or even higher levels of vitamins underscore the significance of a cautious approach in addressing nutritional requirements, including Phe-free products, low-protein foods, and vitamin supplements, as well as considering not only deficiencies but also excesses of vitamins. Future research should explore the impact of specific treatment regimens and compliance in PKU patients on vitamin status.

## Figures and Tables

**Table 1:** Characteristics of included studies and studied participants.

Author	Year	Country (Region)	Study Design	Groups	*n* Included	*n* Completed	Age [Years] ^1^	BMI [kg/m^2^] ^1^	Sex [% of Women]
Rojas-Agurto et al. [39]	2023	Chile	Cross-sectional	PKU ^2^	10	10	23.5 (19–26) ^4^	24.3 (22.4–28.5) ^4^	50
				Control	10	10	21.5 (20–27) ^4^	24.3 (24.1–27.9) ^4^	50
				PKU ^3^	14	14	22.5 (18.5–25.5) ^4^	26.7 (24–29.9) ^4^	36
				Control	14	14	23 (19–25) ^4^	27.6 (23.3–30.6) ^4^	36
Leiva et al. [47]	2021	Chile	Cross-sectional	PKU ^2^	16	16	6–23 ^5^	NI	44
				Control	16	16	NI	NI	44
Akış et al. [42]	2020	Turkey	Cross-sectional	PKU ^6^	31	31	9.50 (5.00–18.00) ^8^	NI	38
				PKU ^7^	22	22			
				Control	30	30	8.59 (5.00–17.50) ^8^	NI	53
Ekin et al. [31]	2018	Turkey	Cross-sectional	PKU ^2^	30	30	7.54 ± 0.58 ^9^	16.16 ± 0.54 ^9^	60
				Control	30	30	7.89 ± 0.74 ^9^	17.64 ± 0.53 ^9^	56.6
Veyrat-Durebex et al. [52]	2017	France	Cross-sectional	PKU ^10^	10	9	31 ± 6	26 ± 4	60
				Control	10	9	NI	22 ± 4	NI
Gündüz et al. [43]	2016	Turkey	Cross-sectional	PKU ^11^	24	24	13.1 ± 2.4	19.1 ± 2.1	33
				PKU ^12^	35	35	14.1± 2.9	18.9± 1.9	54
				Control	44	44	13.0 ± 2.0	19.7± 2.0	57
Nagasaka et al. [29]	2013	Japan	Cross-sectional	PKU ^13^	33	33	28.1 ± 5.1	23.7 ± 2.2	54.5
				Control	20	20	28.9 ± 4.5	23.1 ± 1.9	50
Mikoluc et al. [28]	2012	Poland	Cross-sectional	PKU ^14^	107	107 ^15^	8.8 ± 2.06	NI	43
				Control	62	62	8.6 ± 1.1	NI	50
Mütze et al. [51]	2012	Germany	Cross-sectional	PKU ^11^	12	12	7.88 (5–14) ^8^	16.68/0.6 (14.51/−1.24–23.21/1.51) ^8,16^	50
				Control	8	8	9.75 (5–17) ^8^	16.22/−0.1 (13.15/−1.71–22.41/1.18) ^8,16^	62.5
Huemer et al. [46]	2012	Austria	Cross-sectional	PKU ^2^	16	16	10.1 ± 5.2	19.5 ± 4.5	43.75
				Control	91	91	11.6 ± 3.7	19.2 ± 4.3	35
Nagasaka et al. [30]	2011	Japan	Cross-sectional	PKU ^17,18^	21	21	27.1 ± 3.2 ^9^	22.5 ± 1.5 ^9^	100
				PKU ^17,19^	13	13	26.9 ± 3.3 ^9^	24.7 ± 2.4 ^9^	0
				Control ^18^	22	22	27.9 ± 5.1 ^9^	23.7 ± 2.2 ^9^	100
				Control ^19^	14	14	30.3 ± 4.5 ^9^	23.5 ± 2.3 ^9^	0
Huemer et al. [26]	2008	Austria	Case-control	PKU ^2^	37	37	12.3 ± 4.5	19.1 ± 3.3	35.1
				Control	63	63	11.5 ± 4.1	NI	41.3
Gassió et al. [32]	2008	Spain	Cross-sectional	PKU ^2^	36	36	9.7 (2.7–19.4) ^20^	NI	47.2
				Control	29	29	9.6 (2.5–18.8) ^20^	NI	48.3
Colomé et al. [44]	2003	Spain	Cross-sectional	PKU ^2,21^	42	42	15.3 ± 9.5	NI	NI
				Control	42	42	15.7 ± 9.7	NI	NI
Schulpis et al. [25]	2003	Greece	Cross-sectional	PKU ^11^	22	22	7.7 ± 3.2	NI	NI
				PKU ^12^	24	24	8.0 ± 3.6	NI	NI
				Control	40	40	7.68 ± 2.6	NI	NI
Schulpis et al. [27]	2002	Greece	Cross-sectional	PKU ^11^	34	34	6.78 ± 1.5	NI	NI
				PKU ^12^	40	40	8.0 ± 3.2	NI	NI
				Control	50	50	7.68 ± 2.3	NI	NI
Lucock et al. [48]	2002	The United Kingdom	Cross-sectional	PKU	16	16	26.5 (17–37) ^8^	NI	NI
				Control	25	25	33 (25–53) ^8^	NI	NI
van Bakel et al. [53]	2000	Switzerland	Cross-sectional	PKU ^22^	24	24	9.65 ± 4.06	NI	41.7
				PKU ^23^	10	10	9.08 ± 5.17	NI	50
				Control	42	42	11.18 ± 4.84	NI	28.6
Robinson et al. [49]	2000	The United Kingdom	Cross-sectional	PKU ^24^	22	22 ^26^	24 ^29^	NI	NI
				PKU ^25^	30	30 ^27^	21 ^29^	NI	NI
				Control	1676	1676 ^28^	NI	NI	NI
Al-Qadreh et al. [41]	1998	Greece	Cross-sectional	PKU	48	48	8.86 ± 3.7	NI	58.3
				Control	50	50	9.06 ± 3.5	NI	58.3
Schulpis et al. [40]	1998	Greece	Cross-sectional	PKU ^11^	21	21	4.78 ± 3.51	NI	NI
				PKU ^12^	26	26	7.87 ± 3.68	NI	NI
				Control	79	79	6.68 ± 2.3	NI	NI
Sierra et al. [45]	1998	Spain	Cross-sectional	PKU ^11^	42	42	7.12 (1 month–17 years) ^20^	NI	NI
				Control	45	45	6.5 (1 month–17 years) ^20^	NI	NI
Hillman et al. [50]	1996	The USA	Cross-sectional	PKU	11	11	10.9 ± 4.2	NI	54.5
				Control	64	64 ^30,31^	11.4 ± 4.2	NI	50
Prince et al. [38]	1994	The USA	Cross-sectional	PKU ^2^	16	15	10.5 ± 2.9	NI	40
				Control	6	6	9.4 ± 3.3	NI	33.3

^1^—mean ± standard deviation; ^2^—patients under diet treatment; ^3^—patients who discontinued the protein substitution at 18 years of age; ^4^—median (25th–75th centile); ^5^—range; ^6^—patients with high adherence; ^7^—patients with low adherence; ^8^—median (min–max); ^9^—mean ± standard error of the mean; ^10^—patients followed a Phe-restrictive diet in childhood and stopped it at the average age of 6 years; ^11^—well-controlled; ^12^—poorly controlled; ^13^—patients exclusively received phenylalanine-restricted diets; after the age of 20 years, restrictions of phenylalanine differed greatly among patients; ^14^—patients keeping phenylalanine levels in the blood within the recommended values; ^15^—vitamin analyses were performed in 99 participants; ^16^—BMI/SDS (standard deviation score); ^17^—until the age of 15 years, patients received phenylalanine-restricted diets with phenylalanine-free milk; thereafter, the restrictions of phenylalanine varied among the patients; ^18^—female participants; ^19^—male participants; ^20^—mean (range); ^21^—27 had acceptable dietary control and 15 had poor dietary compliance; ^22^—phenylalanine tolerance < 12 mg phenylalanine·kg^−1^·d^−1^; ^23^—phenylalanine tolerance > 12 mg phenylalanine·kg^−1^·d^−1^); ^24^—strict diet; ^25^—relaxed diet; ^26^—folate concentration analysis was performed in 11 participants; ^27^—folate concentration analysis was performed in 21 participants; ^28^—folate concentration data were obtained from 1502 individuals; ^29^—median; ^30^—25(OH)D vitamin concentration analysis was performed in 29 participants; and ^31^—1,25(OH)_2_D vitamin concentration analysis was performed in 27 participants. BMI—body mass index; PKU—phenylketonuria; NI—no information.

**Table 2:** Characteristics of diet and metabolic status of studied individuals.

Author	Year	Groups	Natural Protein/Protein Substitute/Total Protein Intake [g/day] ^1^	Phe Intake [mg/d]	Mean/Median Phe Levels [μmol/L] ^1^	Medical Control	Adherence to Treatment	Phe [μmol/L] ^1^	Tyr [μmol/L] ^1^	Total Protein Level [g/dL]
Rojas-Agurto et al. [39]	2023	PKU ^2^	75.1 (57.3–78.2) ^4^	600 (400–800) ^4^	NI	Yes	Yes	260.3 (170–642) ^4^	46.6 (33.1–49.7) ^4^	NI
		Control	84.4 (57.9–101) ^4^	3900 (2600–4900) ^4^		///	///	39.3 (36.3–42.4) ^4^	49.7 (44.2–60.7) ^4^	
		PKU ^3^	46.6 (28.9–68.7) ^4^	1200 (500–1700) ^4^		No	No	781 (636–1035.1) ^4^	35.9 (33.1–55.2) ^4^	
		Control	89.6 (66.6–101.1) ^4^	4000 (3100–4500) ^4^		///	///	47.8 (40.6–48.4) ^4^	53 (44.2–60.7) ^4^	
Leiva et al. [47]	2021	PKU ^2^	NI	NI	NI	Yes	Yes	310 (262; 481) ^5^	NI	NI
		Control				///	///	NI		
Akış et al. [42]	2020	PKU ^6^	NI	NI	NI	Yes	High	357.19 (121.10–514.60) ^8^	NI	NI
		PKU ^7^				Yes	Low	696.21 (441.90–1035.20) ^8^	NI	NI
		Control				///	///	NI	NI	NI
Ekin et al. [31]	2018	PKU ^2^	NI	NI	NI	NI	NI	269.50 ± 49.75 ^9^	59.62 ± 10.58 ^9^	NI
		Control				///	///	51.07 ± 3.52 ^9^	57.58 ± 4.45 ^9^	
Veyrat-Durebex et al. [52]	2017	PKU ^10^	NI	NI	NI	NI	No	1449 (363;1854) ^8^	51 (30;56) ^8^	NI
		Control				///	///	60 (50;95) ^8^	69 (45;77) ^8^	
Gündüz et al. [43]	2016	PKU ^11^	0.20–0.40/0.6–0.8/0.8–1.2 ^13^	300–900	NI	NINI	Yes	306.1± 78.0	NI	NI
		PKU ^12^					No	720.8± 196.7		
		Control	NI	NI		///	///	48.8± 12.2		
Nagasaka et al. [29]	2013	PKU ^14^	50 ± 13/ /71 ± 17	NI	NI	NI	NI	1019 ± 380	NI	NI
		Control	66 ± 14/-/80 ± 15			///	///	30 ± 15		
Mikoluc et al. [28]	2012	PKU ^15^	NI	NI	NI	Yes	Yes	NI	NI	NI
		Control				///	///			
Mütze et al. [51]	2012	PKU ^11^	-/-/41.1 (22.5–78.3) ^8^	338 (254–690) ^8^	NI	NI	Yes	302 (72–930) ^8^	NI	7.05 (6.37–7.3) ^8^
		Control	NI	NI	NI	///	///	53 (48–75) ^8^	NI	NI
Huemer et al. [46]	2012	PKU ^2^	0.23 ± 0.11/-/1.3 ± 0.4	NI	NI	NI	NI	809 (400–919) ^4^	NI	NI
		Control	NI			///	///	6250 ± 80 ^4^	NI	NI
Nagasaka et al. [30]	2011	PKU ^16, 17^	40 ± 11/-/66 ± 15	NI	NI	NI	NI	22.3 ± 4.5	NI	7.2 ± 0.3 ^9^
		PKU ^16, 18^	47 ± 10/-/72 ± 17					NI		7.3 ± 0.3 ^9^
		Control ^17^	79 ± 10/-/79 ± 10			///	///	NI		7.5 ± 0.3 ^9^
		Control ^18^	88 ± 11/-/88 ± 11			///	///	NI		7.6 ± 0.3 ^9^
Huemer et al. [26]	2008	PKU ^2^	0.3 ± 0.2//1.8 ± 0.3	NI	NI	NI	NI	620 ± 425	NI	NI
		Control	NI			///	///	///		
Gassió et al. [32]	2008	PKU ^2^	NI	NI	NI	NI	Yes	NI	57.41 ± 24.84	NI
		Control				///	///		72 (53–87) ^19^	
Colomé et al. [44]	2003	PKU ^2, 20^	NI	NI	NI	NI	Mixed group	NI	NI	NI
		Control					///			
Schulpis et al. [25]	2003	PKU ^11^	-/-/70 ± 18 ^21^	NI	292 ± 60 ^22^		Yes	NI	115.3 ± 26.5	NI
		PKU ^12^	-/-/72.0 ± 20 ^21^		895 ± 54 ^22^		No		45.8 ± 27.5	
		Control	-/-/73.0 ± 17 ^21^		NI	///	///		139.6 ± 32.1	
Schulpis et al. [27]	2002	PKU ^11^	6 ± 1.2/-/70 ± 13	NI	NI		Yes	192 ± 115	NI	NI
		PKU ^12^	30 ± 1.6/-/72 ± 14				No	599 ± 16		
		Control	74 ± 15/-/74 ± 15			///	///	70.1 ± 100		
Lucock et al. [48]	2002	PKU	NI	NI	NI		No	1037 (560–1760) ^8^	NI	NI
		Control				///	///	NI		
van Bakel et al. [53]	2000	PKU ^23^	NI	NI	NI	NI	NI	NI	NI	NI
		PKU ^24^								
		Control				///	///			
Robinson et al. [49]	2000	PKU ^25^	NI	NI	NI	Yes	Yes	NI	NI	NI
		PKU ^26^				Yes	No			
		Control				///	///			
Al-Qadreh et al. [41]	1998	PKU	NI	NI	NI	Yes	NI	111.3 ± 66.20	NI	NI
		Control				///	///	///		
Schulpis et al. [40]	1998	PKU ^11^	26.0 ± 7.2/42.0 ± 5.0/68.1 ± 12.9	NI	20 ± 9 ^22^		Yes	31.0 ± 9.0	NI	NI
		PKU ^12^	35.0 ± 8.2/35.0 ± 11.5/70.9 ± 19.7		15.87 ± 5.09 ^22^		No	18.87 ± 3.09		
		Control	-/-/72.3 ± 16.9		NI	///	///	1.2 ± 0.5		
Sierra et al. [45]	1998	PKU ^11^	NI	NI	NI		Yes	NI	NI	NI
		Control				///	///			
Hillman et al. [50]	1996	PKU	-/-/46.1 ± 12.1	NI	99.8 ± 95.4 ^27^	NI	NI	NI	NI	NI
		Control	NI		NI	///	///			
Prince et al. [38]	1994	PKU ^2^	-/-/44.2 ± 10	NI	NI	Yes	No ^28^	882 ± 284	NI	NI
		Control	-/-/69.2 ± 17			///	///	70 ± 13		

^1^—mean ± standard deviation; ^2^—patients under diet treatment; ^3^—patients who discontinued the protein substitution at 18 years of age; ^4^—median (25th–75th centile); ^5^—median and 95% CI; ^6^—patients with high adherence; ^7^—patients with low adherence; ^8^—median (min–max); ^9^—mean ± standard error of the mean; ^10^—patients followed a Phe-restrictive diet in childhood and stopped it at the average age of 6 years; ^11^—well-controlled; ^12^—poorly controlled; ^13^—g/kg/day; ^14^—patients exclusively received phenylalanine-restricted diets; after the age of 20 years, restrictions of phenylalanine differed greatly among patients; ^15^—patients with phenylalanine levels in the blood kept within the recommended values; ^16^—until the age of 15 years, patients received phenylalanine-restricted diets with phenylalanine-free milk; thereafter, the restrictions of phenylalanine varied among the patients; ^17^—female participants; ^18^—male participants; ^19^—mean (range); ^20^—27 had acceptable dietary control and 15 had poor dietary compliance; ^21^—per 100 g; ^22^—mean annual Phe levels; ^23^—phenylalanine tolerance < 12 mg phenylalanine·kg^−1^·d^−1^; ^24^—phenylalanine tolerance > 12 mg phenylalanine·kg^−1^·d^−1^); ^25^—strict diet; ^26^—relaxed diet; ^27^—mean average of three serum Phe values prior to study; and ^28^—nearly all patients had plasma phenylalanine concentrations above the recommended upper safe limit and about one-third of these patients were no longer consuming medical foods regularly. Phe—phenylalanine; Tyr—tyrosine; PKU—phenylketonuria; NI—no information.

**Table 3:** Comparison of vitamin status between PKU individuals and healthy controls.

Author	Year	Groups	Folate ^1^	Vitamin B12 ^1^	Vitamin D ^1^	1,25(OH)_2_D Vitamin ^1^	Vitamin A ^1^	Beta-Carotene ^1^	Vitamin E ^1^	Vitamin B6 ^1^	Vitamin K ^1^	Vitamin C ^1^	Biotin ^1^
Rojas-Agurto et al. [39]	2023	PKU ^2^	25.69 ± 7.58 ^4,5,6,7^	669.2 ± 330.4 ^4,6,7^	36.97 ± 9.33 ^4,7,8,9^	NA	NA	NA	NA	NA	NA	NA	NA
		Control	15.72 ± 5.39 ^4,5,6,7^	454 ± 216.6 ^4,6,7^	29.26 ± 8.98 ^4,7,8,9^								
		PKU ^3^	23.68 ± 7.19 ^4,5,6,7^	383.4 ± 253.2 ^4,6,7^	24.3 ± 10.62 ^4,7,8,9^								
		Control	16.63 ± 4.57 ^4,5,6,7^	444.9 ± 134.8 ^4,6,7^	30.45 ± 10.29 ^4,7,8,9^								
Leiva et al. [47]	2021	PKU ^2^	NA	NA	38.61 ± 8.67 ^4,6,7,8^	NA	NA	NA	NA	NA	NA	NA	NA
		Control			33.84 ± 7.93 ^4,6,7,8^								
Akış et al. [42]	2020	PKU ^10^	36.39 ± 9.85 ^4,7,13^	297.4 ± 126.5 ^4,7,15^	NA	NA	NA	NA	NA	NA	NA	NA	NA
		PKU ^11^	37.05 (15.5–54.6) ^4,13,14^	281.2 (100–607) ^4,14,15^									
		PKU ^12^	36.36 (12.3–54.6) ^4,13,14^	266.8 (117–568) ^4,14,15^									
		Control	22.61 ± 7.38 ^4,7,13,14^	291.4 ± 81.6 ^4,7,14,15^									
Ekin et al. [31]	2018	PKU ^2^	NA	NA	0.43 ± 0.035 ^4,16,17,18^	NA	2.91 ± 0.21 ^4,16,18^	NA	2.26 ± 0.19 ^4,16,18^	NA	0.73 ± 0.078 ^4,16,18^	NA	NA
		Control			0.38 ± 0.033 ^4,16,17,18^		3.03 ± 0.14 ^4,16,18^		2.39 ± 0.16 ^4,16,18^		1.01 ± 0.056 ^4,16,18^		
Veyrat-Durebex et al. [52]	2017	PKU ^19^	NA	NA	NA	NA	1.8 (1.3;3.28) ^14,18,20^	NA	22.4 (18;36) ^14,18,20^	NA	NA	58 (20;90) ^14,18,20^	NA
		Control					1.67 (1;2.03) ^14,18,20^		24.2 (19;32) ^14,18,20^			75 (17;102) ^14,18,20^	
Gündüz et al. [43]	2016	PKU ^21^	32.4± 9.7 ^4,5,13^	256.5± 139.3 ^4,15^	NA	NA	NA	NA	NA	NA	NA	NA	NA
		PKU ^22^	32.8 ± 9.0 ^4,5,13^	308.8± 119.1 ^4,15^									
		Control	22.5± 7.2 ^4,5,13^	303.0± 85.8 ^4,15^									
Nagasaka et al. [29]	2013	PKU ^23^	NA	NA	19.9 ± 2.1 ^4,6,24^	55.5 ± 3.7 ^4,9^	NA	NA	NA	NA	NA	NA	NA
		Control			28.9 ± 2.3 ^4,6,24^	40.7 ± 2.7 ^4,9^							
Mikoluc et al. [28]	2012	PKU ^25^	NA	NA	NA	NA	3.36 ± 2.11 ^18,20^	NA	9.89 ± 7.75 ^18,20^	NA	NA	NA	NA
		Control					2.74 ± 1.93 ^18,20^		25.40 ± 17.52 ^18,20^				
Mütze et al. [51]	2012	PKU ^21^	45.4 (30.3 -> 45.4) ^4,13,14^	774.8 (289.9–1229) ^4,14,15^	NA	NA	NA	NA	NA	NA	NA	NA	NA
		Control	28.1 (15.4–45.2) ^4,13,14^	352.6 (238.6–500) ^4,14,15^									
Huemer et al. [46]	2012	PKU ^2^	7.2 ± 3.2 ^7,13,20^	NA	NA	NA	NA	NA	NA	NA	NA	NA	NA
		Control	6.5 ± 2.9 ^7,13,20^										
Nagasaka et al. [30]	2011	PKU ^26,27^	NA	NA	18.7 ± 1.3 ^4,6,8,16^	58.4 ± 2.7 ^4,9,16^	NA	NA	NA	NA	NA	NA	NA
		PKU ^26,28^			22.2 ± 1.7 ^4,6,8,16^	50.6 ± 2.0 ^4,9,16^							
		Control ^27^			27.6 ± 2.1 ^4,6,8,16^	41.6 ± 3.1 ^4,9,16^							
		Control ^28^			30.0 ± 2.6 ^4,6,8,16^	39.9 ± 2.7 ^4,9,16^							
Huemer et al. [26]	2008	PKU ^2^	15.1 ± 5.1 ^4,6^	783 ± 528 ^4,9^	NA	NA	NA	NA	NA	146 ± 72 ^4,13^	NA	NA	NA
		Control	7.6 ± 3.3 ^4,6^	478 ± 180 ^4,9^						NI			
Gassió et al. [32]	2008	PKU ^2^	NA	NA	NA	NA	1.36 ± 0.38 ^20,29^	NA	20 ± 4.57 ^20,29^	NA	NA	NA	NA
		Control					2.10 ± 4.12 ^20,29^		21 ± 6.74 ^20,29^				
Colomé et al. [44]	2003	PKU ^2,30^	49.1 ± 8.6 ^4,13^	622 ± 253 ^4,15^	NA	NA	NA	NA	NA	NA	NA	NA	NA
		Control	19.1 ± 7.5 ^4,13^	455 ± 161 ^4,15^									
Schulpis et al. [25]	2003	PKU ^21^	NA	NA	NA	NA	NA	0.7 ± 0.09 ^18,20^	34 ± 0.9 ^18,20^	NA	NA	36.3 ± 1.1 ^18,20^	NA
		PKU ^22^						0.49 ± 0.08 ^18,20^	22.0 ± 0.6 ^18,20^			34.5 ± 1.1 ^18,20^	
		Control						0.40 ± 0.09 ^18,20^	24.0 ± 1.6 ^18,20^			38.2 ± 2.01 ^18,20^	
Schulpis et al. [27]	2002	PKU ^21^	2.35 ± 1.3 ^4,13^	98.5 ± 22.3 ^4,15^	NA	NA	NA	NA	NA	10.7 ± 0.9 ^4,13^	NA	NA	NA
		PKU ^22,31^	5.8 ± 2.1 ^4,13^	240.8 ± 62 ^4,15^						58.8 ± 9.8 ^4,13^			
		Control	6.1 ± 2.0 ^4,13^	251 ± 68 ^4,15^						60.2 ± 10 ^4,13^			
Lucock et al. [48]	2002	PKU	469.1 (397.7–637.1) ^4,6,32,33^	NA	NA	NA	NA	NA	NA	NA	NA	NA	NA
		Control	363.9 (325.3–457.9) ^4,6,32,33^	NA	NA	NA	NA	NA	NA	NA	NA	NA	NA
van Bakel et al. [53]	2000	PKU ^34^	NA	NA	NA	NA	NA	NA	21.46 ± 4.06 ^18,20^	NA	NA	NA	NA
		PKU ^35^							19.25 ± 2.11 ^18,20^				
		Control							20.93 ± 6.15 ^18,20^				
Robinson et al. [49]	2000	PKU ^36^	476 ± 258 ^32,38^	468.7 ± 199.7 ^39,40^	NA	NA	NA	NA	NA	NA	NA	NA	NA
		PKU ^37^	471 ± 190.5 ^32,38^	332.8 ± 128 ^39,40^									
		Control	201 ± 92.8 ^32,38^	411.9 ± 148.75 ^39,40^									
Al-Qadreh et al. [41]	1998	PKU	NA	NA	45.3 ±3.8 ^4,8,13,16^	NA	NA	NA	NA	NA	NA	NA	NA
		Control			49.16 ±2.54 ^4,8,13,16^								
Schulpis et al. [40]	1998	PKU ^21^	NA	NA	NA	NA	NA	NA	NA	NA	NA	NA	636 ± 118 ^4,40^
		PKU ^22^											411.9 ± 184.9 ^4,40^
		Control											336.6 ± 290.6 ^4,40^
Sierra et al. [45]	1998	PKU ^21^	NA	NA	NA	NA	NA	NA	30.6 ± 9.31 ^29,41^	NA	NA	NA	NA
		Control							26.07 ± 5.43 ^29,41^				
Hillman et al. [50]	1996	PKU	NA	NA	28.3 ± 9.8 ^4,8,42^	36.6 ± 6.7 ^4,42^	NA	NA	NA	NA	NA	NA	NA
		Control			22.3 ± 8.5 ^4,8,42^	30.4 ± 9.3 ^4,42^							
Prince et al. [38]	1994	PKU ^2^	NA	NA	NA	NA	NA	NA	NA	99.4 ± 54 ^13,20^	NA	NA	NA
		Control								53.5 ± 21.3 ^13,20^			

^1^—mean ± standard deviation; ^2^—patients under diet treatment; ^3^—patients who discontinued the protein substitution at 18 years of age; ^4^—serum; ^5^—folic acid; ^6^—ng/mL; ^7^—data were received from authors; ^8^—25(OH)D; ^9^—pg/mL; ^10^—all PKU patients; ^11^—patients with high adherence; ^12^—patients with low adherence; ^13^—nmol/L; ^14^—median (min–max); ^15^—pmol/L; ^16^—mean ± standard error of the mean; ^17^—cholecalciferol; ^18^—µmol/L; ^19^—patients followed a Phe-restrictive diet in childhood and stopped it at the average age of 6 years; ^20^—plasma; ^21^—well-controlled; ^22^—poorly controlled; ^23^—patients exclusively received phenylalanine-restricted diets; after the age of 20 years, restrictions of phenylalanine differed greatly among patients; ^24^—25(OH)D3; ^25^—patients with phenylalanine levels in the blood kept within the recommended values; ^26^—until the age of 15 years, patients received phenylalanine-restricted diets with phenylalanine-free milk; thereafter, the restrictions of phenylalanine varied among the patients; ^27^—female participants; ^28^—male participants; ^29^—data presented in nmol/g prot; ^30^—27 had acceptable dietary control and 15 had poor dietary compliance; ^31^—patients were requested to discontinue their vitamin supplementation for 30 days before the study; ^32^—red blood cells folate; ^33^—median (interquartile range); ^34^—phenylalanine tolerance < 12 mg phenylalanine·kg^−1^·d^−1^; ^35^—phenylalanine tolerance > 12 mg phenylalanine·kg^−1^·d^−1^); ^36^—strict diet; ^37^—relaxed diet; ^38^—µg/L; ^39^—no info about measurement material; ^40^—ng/L; ^41^—erythrocyte tocopherol; and ^42^—no info about measurement unit. PKU—phenylketonuria; NA—not analysed; NI—no information.

**Table 4:** Relative differences between groups.

Study Name	Year	PKU Group Mean	Control Group Mean	Relative Difference
Folate				
Rojas-Agurto et al. [39]	2023	24.52 ^1,2,3,4^	16.25 ^1,2,3,4^	51%
Akış et al. [42]	2020	36.39 ^1,5^	22.61 ^1,5^	61%
Gündüz et al. [43]	2016	32.64 ^1,3,4,5^	22.5 ^1,3,5^	45%
Huemer et al. [46]	2012	7.2 ^5,6^	6.5 ^5,6^	11%
Huemer et al. [26]	2008	15.1 ^1,2^	7.6 ^1,2^	99%
Colomé et al. [44]	2003	49.1 ^1,5^	19.1 ^1,5^	157%
Schulpis et al. [27]	2002	4.21 ^1,4,5^	6.1 ^1,5^	−31%
Lucock et al. [48]	2002	469.1 ^1,2,7^	363.9 ^1,2,7^	29%
Robinson et al. [49]	2000	472.72 ^4,7,8^	201 ^7,8^	135%
Vitamin B12				
Rojas-Agurto et al. [39]	2023	518 ^1,2,4^	448.7 ^1,2^	15%
Akış et al. [42]	2020	297.4 ^1,9^	291.4 ^1,9^	2%
Gündüz et al. [43]	2016	287.5 ^1,4,9^	303 ^1,9^	−5%
Huemer et al. [26]	2008	783 ^1,10^	478 ^1,10^	64%
Colomé et al. [44]	2003	622 ^1,9^	455 ^1,9^	37%
Schulpis et al. [27]	2002	175.4 ^1,9^	251 ^1,9^	−30%
Robinson et al. [49]	2000	390.3 ^4,11,12^	411.9 ^11,12^	−5%
Vitamin D				
Rojas-Agurto et al. [39]	2023	29.58 ^1,4,10,13^	29.95 ^1,10,13^	−1%
Leiva et al. [47]	2021	38.61 ^1,2,13^	33.84 ^1,2,13^	14%
Ekin et al. [31]	2018	0.43 ^1,14,15^	0.38 ^1,14,15^	13%
Nagasaka et al. [29]	2013	19.9 ^1,2,16^	28.9 ^1,2,16^	−31%
Nagasaka et al. [30]	2011	20.04 ^1,2,4,16^	28.53 ^1,2,4,16^	−30%
Al-Qadreh et al. [41]	1998	45.3 ^1,4,13^	49.16 ^1,4,13^	−8%
Hillman et al.	1996	28.3 ^1,13,17^	22.3 ^1,13,17^	27%
1,25-dihydroxyvitamin D				
Nagasaka et al. [29]	2013	55.5 ^1,10^	40.7 ^1,10^	36%
Nagasaka et al. [30]	2011	55.42 ^1,10^	40.94 ^1,10^	35%
Hillman et al. [50]	1996	36.6 ^1,17^	30.4 ^1,17^	20%
		Vitamin A		
Ekin et al. [31]	2018	2.91 ^1,15^	3.03 ^1,15^	−4%
Mikoluc et al. [28]	2012	3.36 ^6,15^	2.74 ^6,15^	23%
Gassió et al. [32]	2008	1.36 ^6,18^	2.1 ^6,18^	−35%
Vitamin E				
Ekin et al. [31]	2018	2.26 ^1,15^	2.39 ^1,15^	−5%
Mikoluc et al. [28]	2012	9.89 ^6,15^	25.4 ^6,15^	−61%
Gassió et al. [32]	2008	20 ^6,18^	21 ^6,18^	−5%
Schulpis et al. [25]	2003	27.74 ^4,6,15^	24 ^6,15^	16%
van Bakel et al. [53]	2000	20.81 ^4,6,15^	20.93 ^6,15^	−1%
Sierra et al. [45]	1998	30.6 ^18,19^	26.07 ^18,19^	17%
		Vitamin B6		
Schulpis et al. [27]	2002	36.7 ^1,4,5^	60.2 ^1,5^	−39%
Prince et al. [38]	1994	99.4 ^5,6^	53.5 ^5,6^	86%

^1^—serum; ^2^—ng/mL; ^3^—folic acid; ^4^—merged values from all groups; ^5^—nmol/L; ^6^—plasma; ^7^—red blood cells folate; ^8^—µg/L; ^9^—pmol/L; ^10^—pg/mL; ^11^—no info about measurement material; ^12^—ng/L; ^13^—25(OH)D; ^14^—cholecalciferol; ^15^—µmol/L; ^16^—25(OH)D3; ^17^—no info about measurement unit; ^18^—data presented in nmol/g prot; and ^19^—erythrocyte tocopherol.

**Table 5:** Newcastle–Ottawa quality assessment scale.

Study (First Author)	Selection					Comparability	Outcome		Overall Score
Cross-Sectional Studies	Representativeness of the Sample	Sample Size	Non- Respondents		Ascertainment of Exposure	Based on Design and Analysis	Assessment of Outcome	Statistical Test	
Rojas-Agurto et al. [39], 2023			+		+ +	+ +			5
Leiva et al. [47], 2021	+	+	+			+ +		+	6
Akış et al. [42], 2020			+		+ +	+ +			5
Ekin et al. [31], 2018			+		+ +	+ +			5
Veyrat-Durebex et al. [52], 2017			+		+ +	+ +			5
Gündüz et al. [43], 2016			+		+ +	+ +			5
Nagasaka et al. [29], 2013			+		+ +	+ +			5
Mikoluc et al. [28], 2012			+		+ +			+	4
Mütze et al. [51], 2012			+		+ +	+ +			5
Huemer et al. [46], 2012			+		+ +				3
Nagasaka et al. [30], 2011			+		+ +	+ +			5
Gassió et al. [32], 2008	+		+		+ +	+ +			6
Colomé et al. [44], 2003	+		+		+ +	+			5
Schulpis et al. [25], 2003			+		+ +	+ +			5
Schulpis et al. [27], 2002			+		+ +	+ +			5
Lucock et al. [48], 2002			+		+ +				3
van Bakel et al. [53], 2000			+		+ +				3
Robinson et al. [49], 2000									0
Al-Qadreh et al. [41], 1998			+						1
Schulpis et al. [40], 1998			+		+ +	+ +			5
Sierra et al. [45], 1998					+ +	+			3
Hillman et al. [50], 1996					+ +	+ +			4
Prince et al. [38], 1994					+ +				2
**Study (first author)**	**Selection**				**Comparability**	**Exposure**			
**Case-control study**	**Case definition adequate?**	**Representativeness of the cases**	**Selection of controls**	**Definition of controls**	**Based on design and analysis**	**Ascertainment of exposure**	**Same method for cases and controls**	**Non-response rate**	**Overall score**
Huemer et al. [26], 2008	+		+		+ +		+	+	6

## Data Availability

Data are available from the corresponding author upon reasonable request.

## Supplementary Materials

The following supporting information can be downloaded at: https://www.mdpi.com/article/10.3390/ijms25105065/s1.

## Abbreviations

BMI—body mass index; CI—confidence interval; NA—not analysed; NI—no information; NOS—Newcastle-Ottawa Scale; PAH—phenylalanine hydroxylase gene; Phe—phenylalanine; PKU—phenylketonuria; SD—standard deviation; SMD—standardised mean difference; Tyr—tyrosine.
