# Supplementary figures and images for: Vitamin Status in Patients with Phenylketonuria: A Systematic Review and Meta-Analysis
Source: Int J Mol Sci. 2024 May 7;25(10):5065. doi: 10.3390/ijms25105065 (PMC11120668; doi:10.3390/ijms25105065)

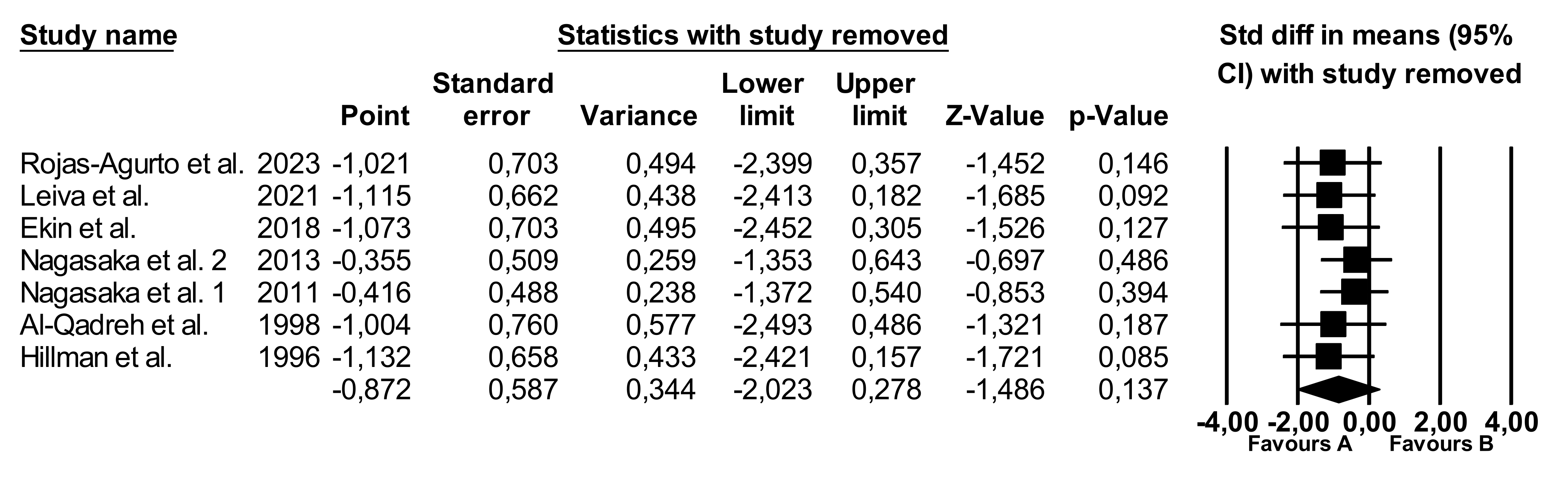

Supplement: Supplementary file 1 [file ijms-25-05065-s001.zip › Figure S10. Sensitivity for vitamin D.jpg]

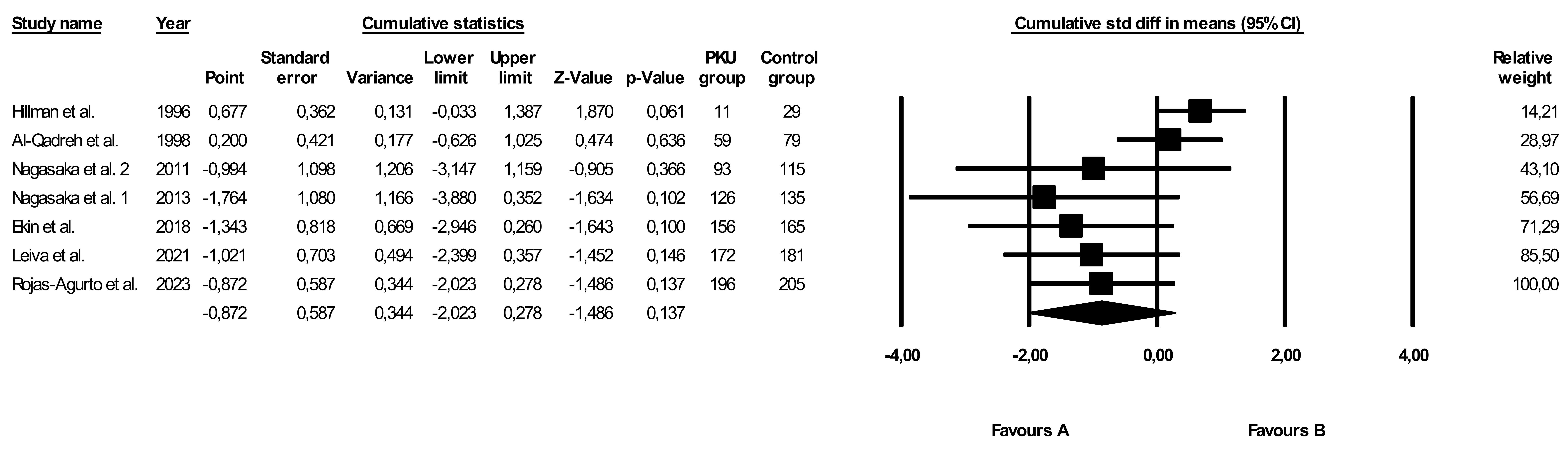

Supplement: Supplementary file 1 [file ijms-25-05065-s001.zip › Figure S11. Cumulative for vitamin D.jpg]

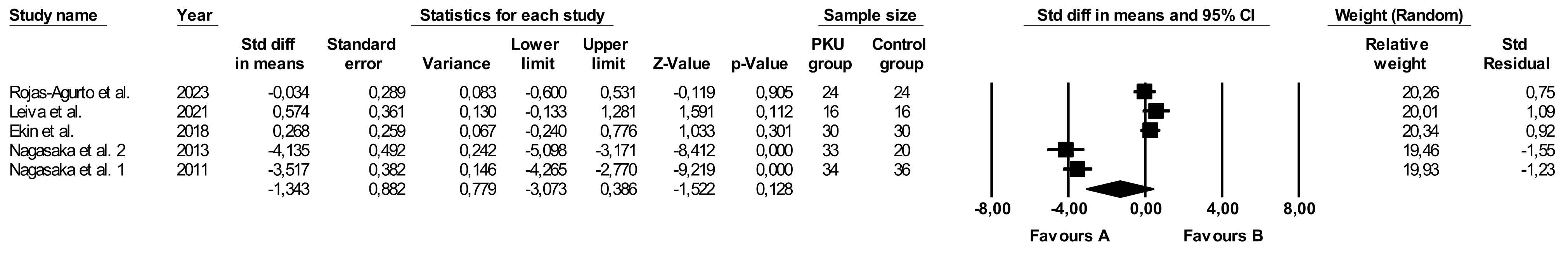

Supplement: Supplementary file 1 [file ijms-25-05065-s001.zip › Figure S12. D without hrob.jpg]

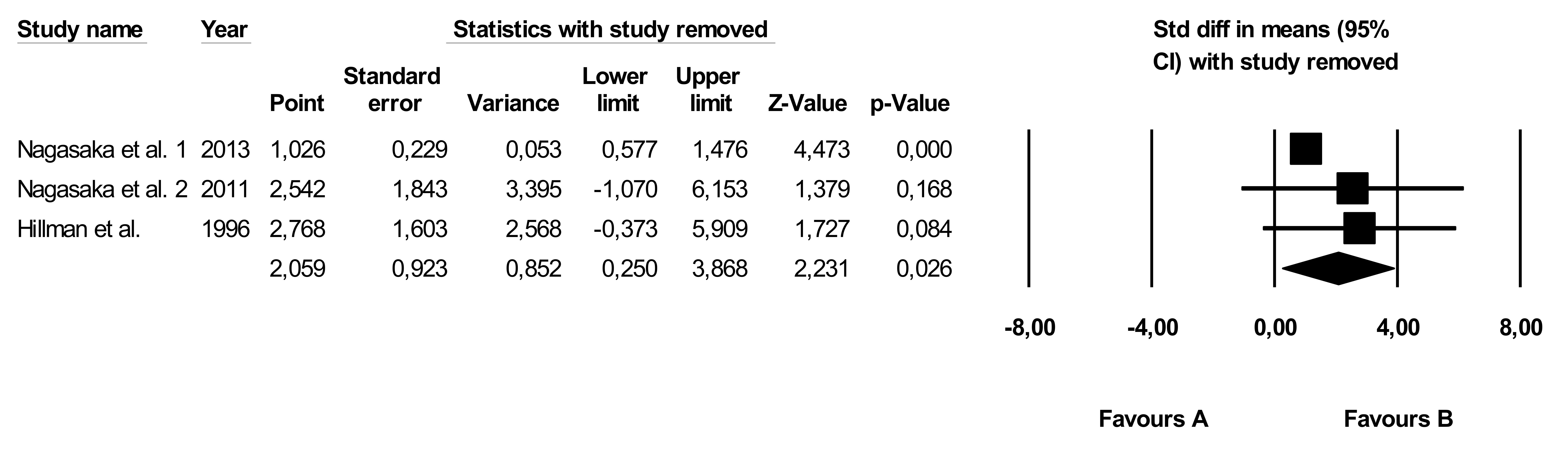

Supplement: Supplementary file 1 [file ijms-25-05065-s001.zip › Figure S14. Sensitivity for 1.25 vitamin D.jpg]

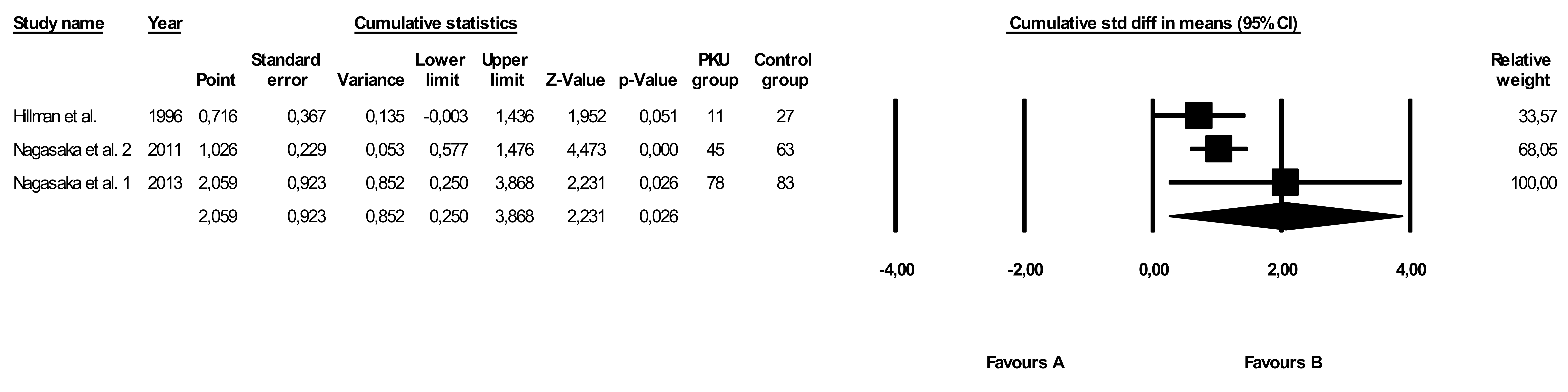

Supplement: Supplementary file 1 [file ijms-25-05065-s001.zip › Figure S15. Cumulative for 1.25 vitamin D.jpg]

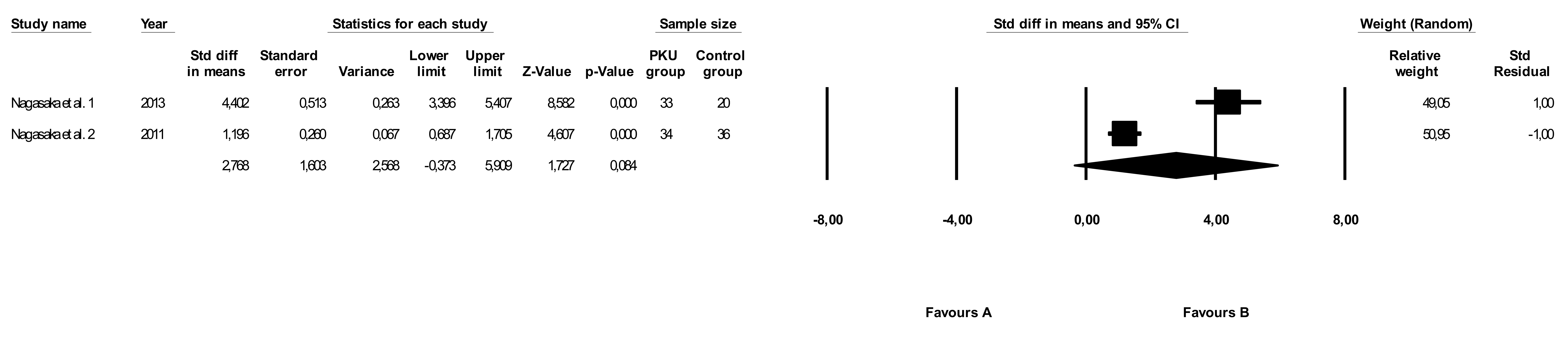

Supplement: Supplementary file 1 [file ijms-25-05065-s001.zip › Figure S16. 1.25 vitamin D without hrob.jpg]

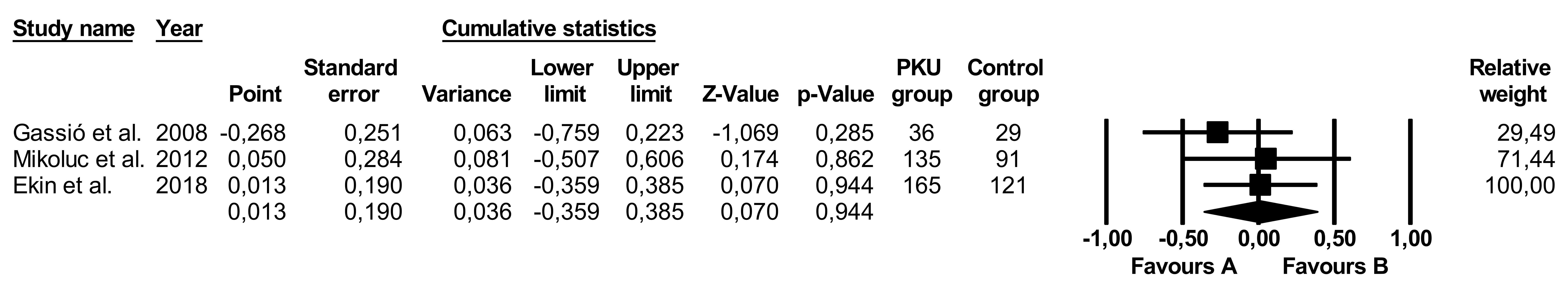

Supplement: Supplementary file 1 [file ijms-25-05065-s001.zip › Figure S18. Cumulative for vitamin A.jpg]

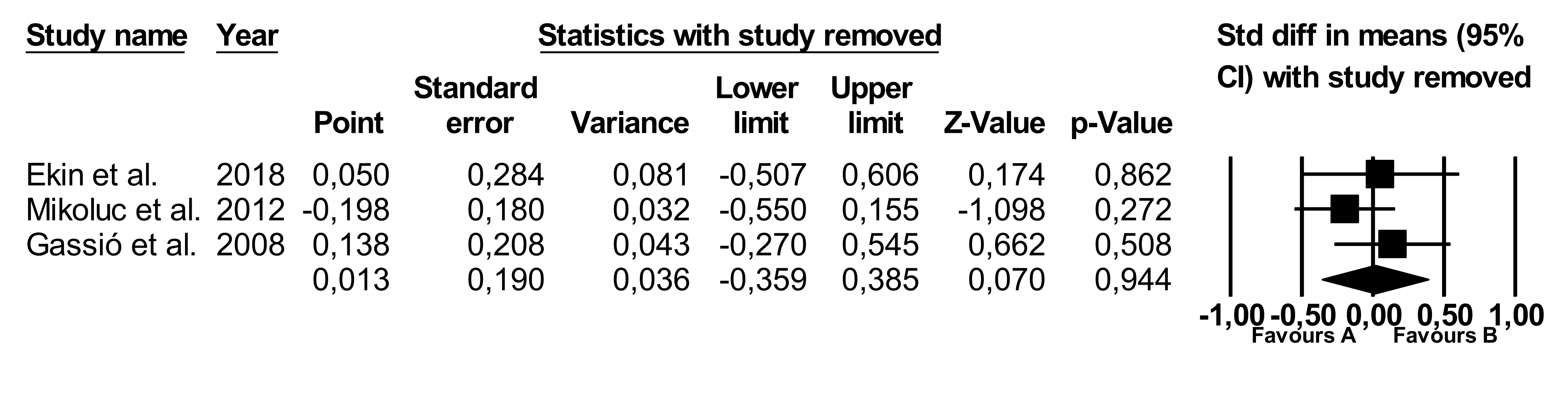

Supplement: Supplementary file 1 [file ijms-25-05065-s001.zip › Figure S19. Sensitivity for vitamin A.jpg]

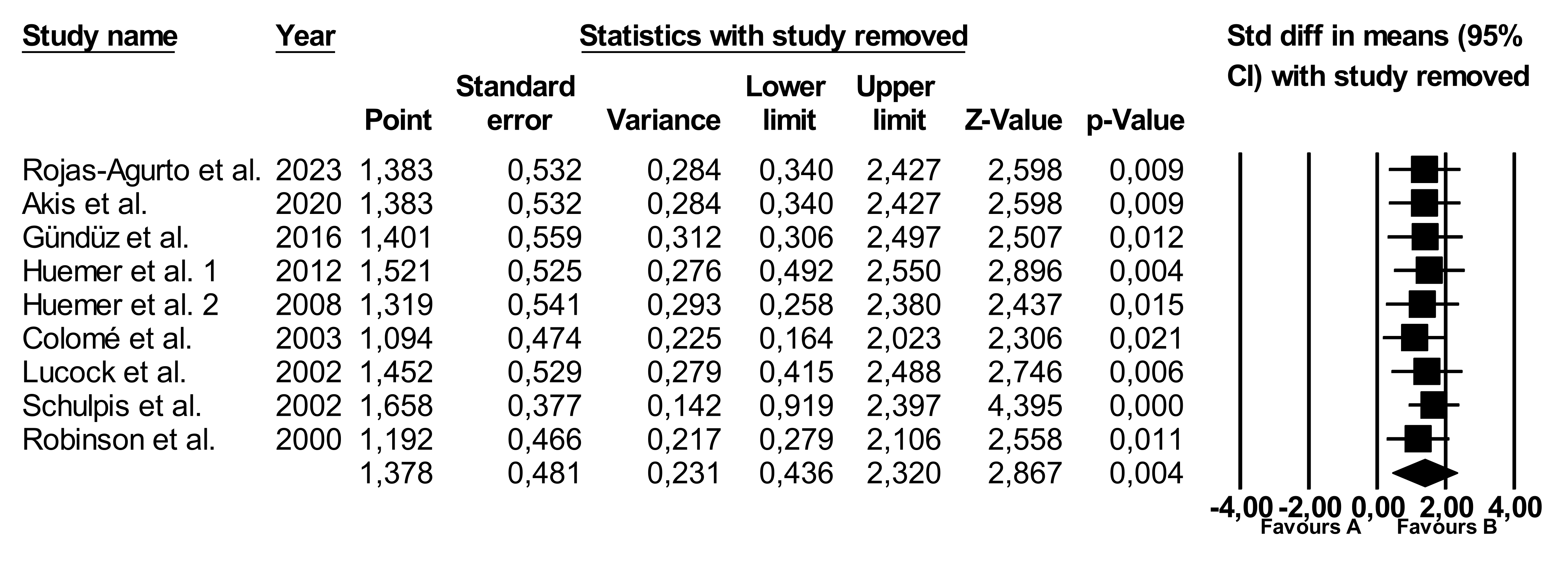

Supplement: Supplementary file 1 [file ijms-25-05065-s001.zip › Figure S2. Sensitivity for folate.jpg]

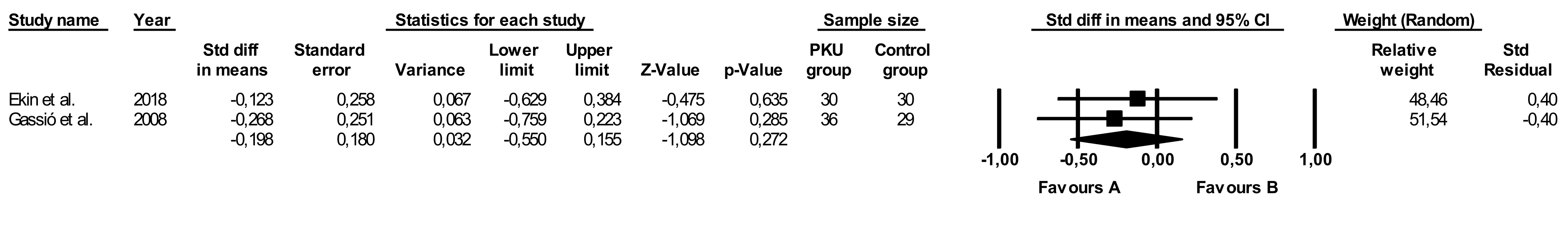

Supplement: Supplementary file 1 [file ijms-25-05065-s001.zip › Figure S20. Vit A without hrob.jpg]

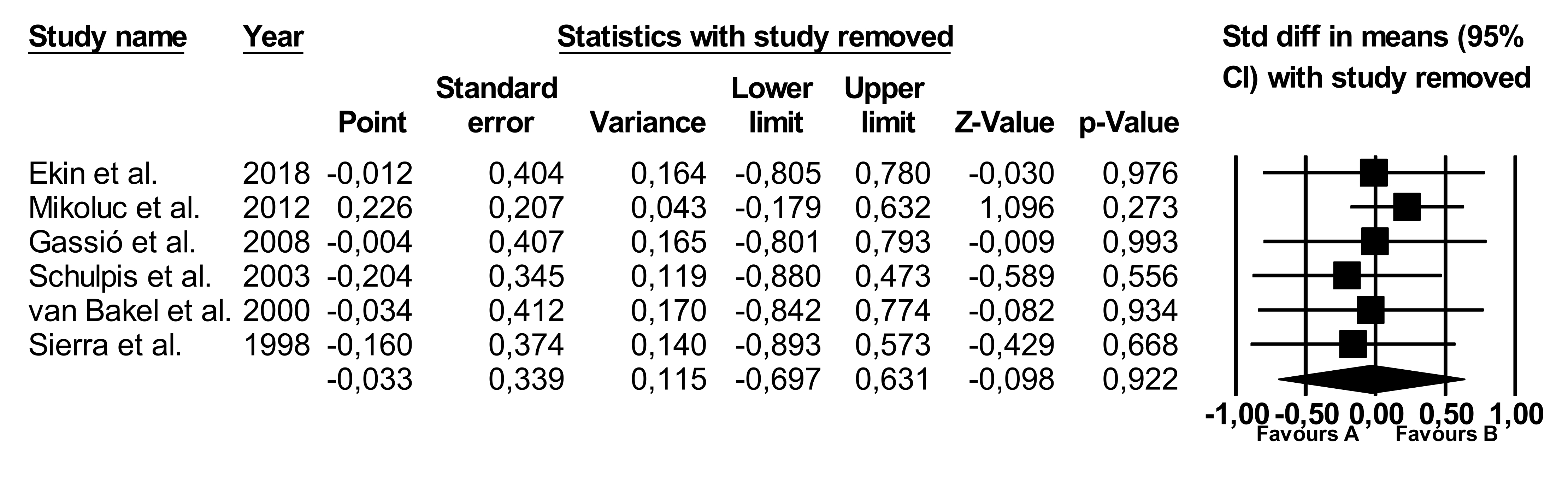

Supplement: Supplementary file 1 [file ijms-25-05065-s001.zip › Figure S22. Sensitivity for vitamin E.jpg]

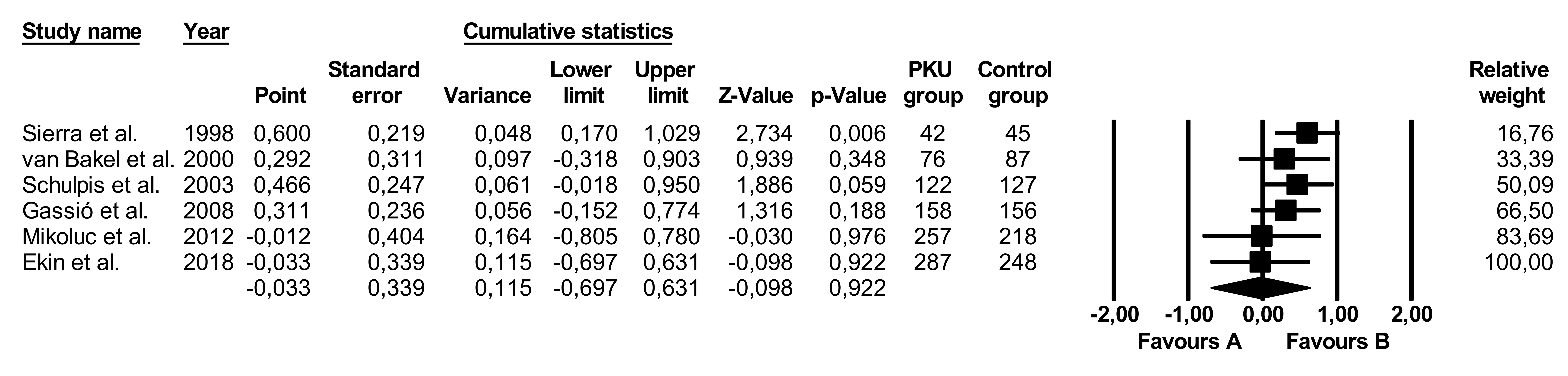

Supplement: Supplementary file 1 [file ijms-25-05065-s001.zip › Figure S23. Cumulative for vitamin E.jpg]

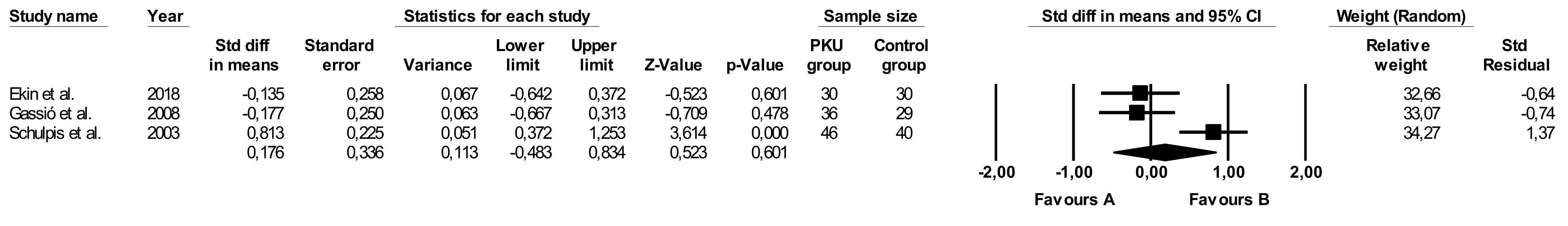

Supplement: Supplementary file 1 [file ijms-25-05065-s001.zip › Figure S24. Vit E without hrob.jpg]

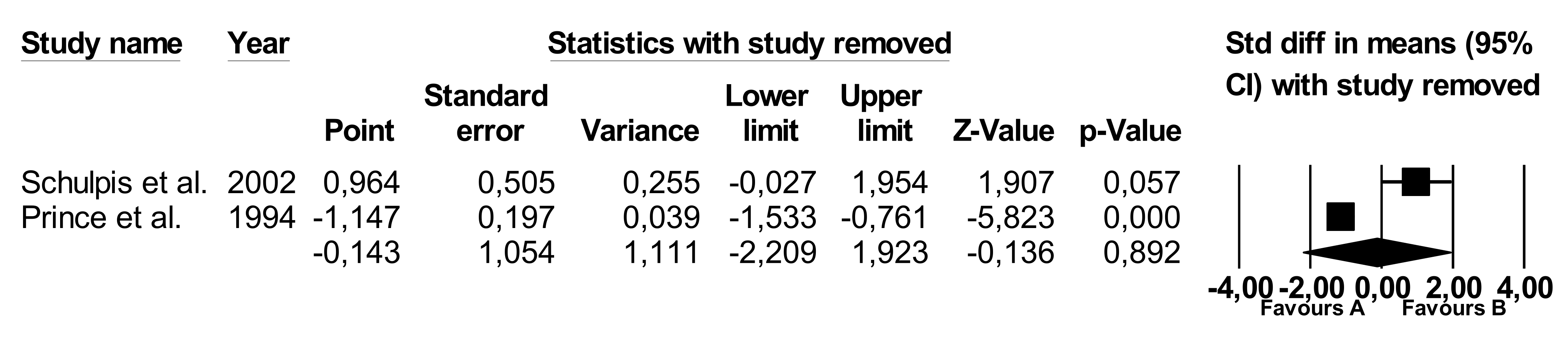

Supplement: Supplementary file 1 [file ijms-25-05065-s001.zip › Figure S25. Sensitivity for vitamin B6.jpg]

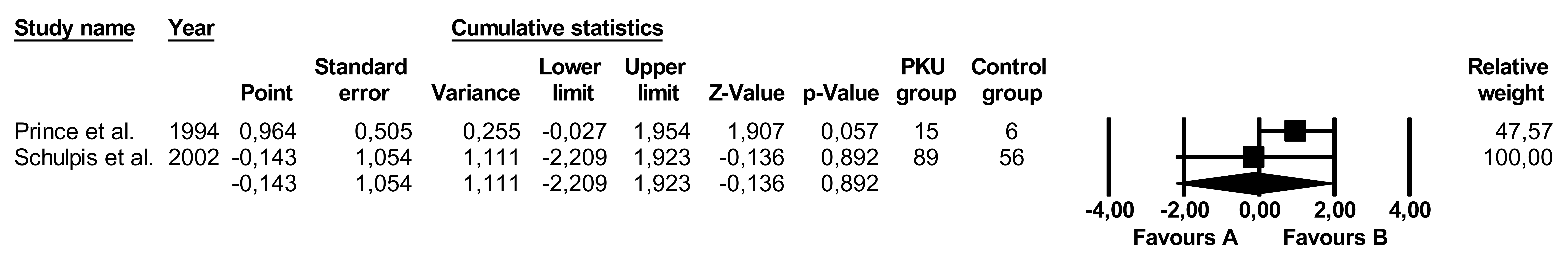

Supplement: Supplementary file 1 [file ijms-25-05065-s001.zip › Figure S26. Cumulative for vitamin B6.jpg]

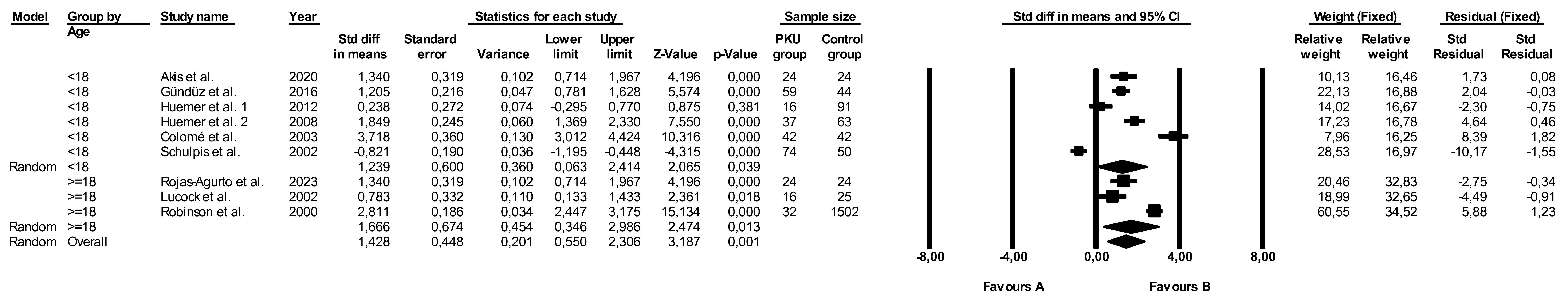

Supplement: Supplementary file 1 [file ijms-25-05065-s001.zip › Figure S27. Folate - age subgroup.jpg]

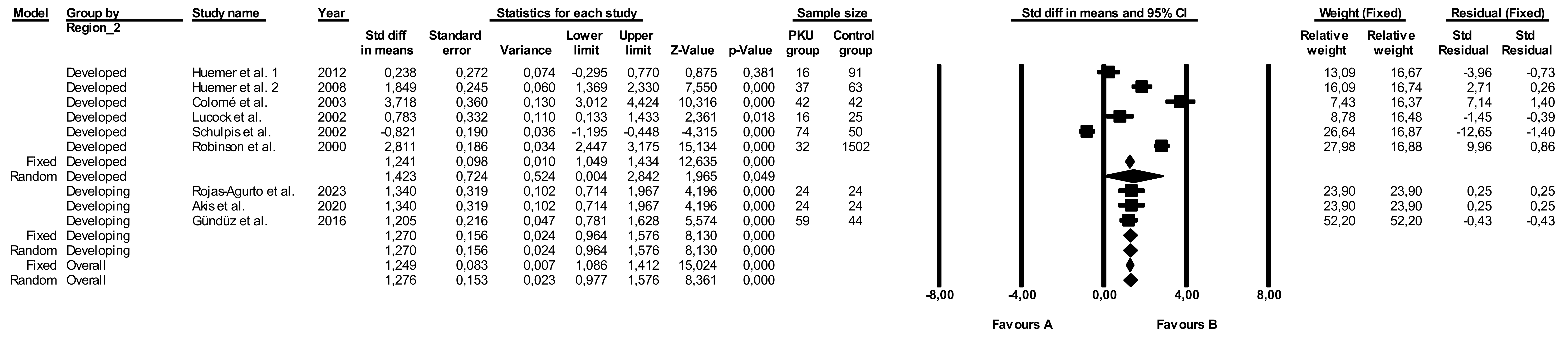

Supplement: Supplementary file 1 [file ijms-25-05065-s001.zip › Figure S28. Folate - country subgroup.jpg]

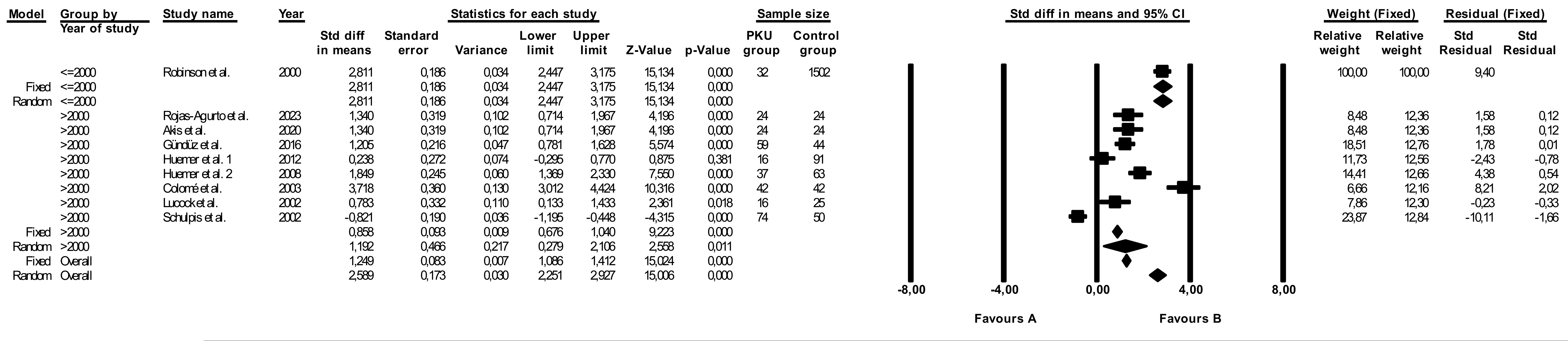

Supplement: Supplementary file 1 [file ijms-25-05065-s001.zip › Figure S29. Folate - year subgroup.jpg]

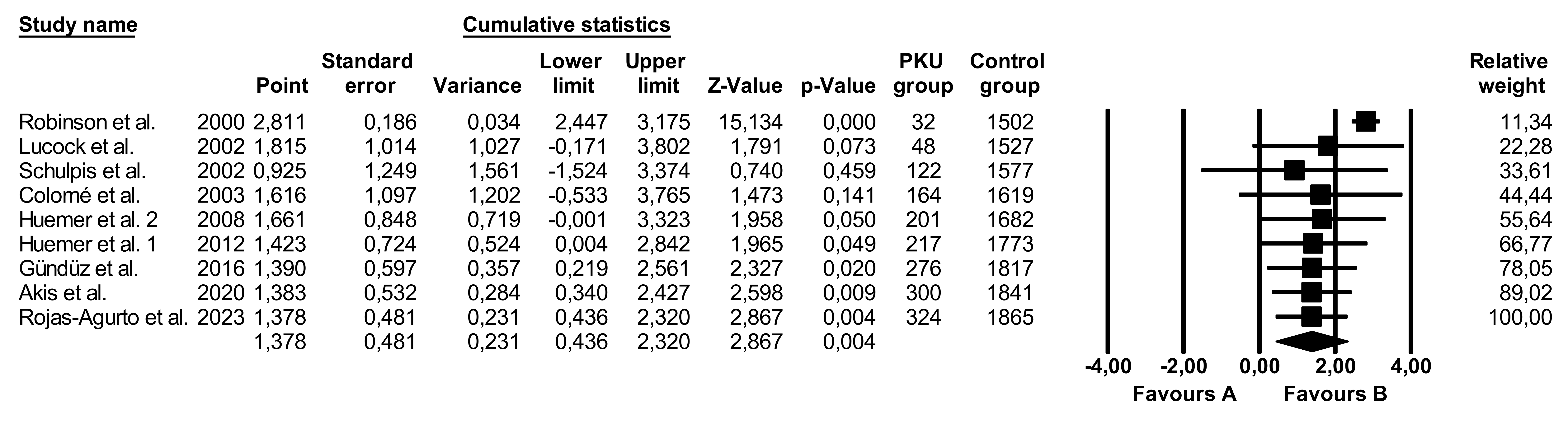

Supplement: Supplementary file 1 [file ijms-25-05065-s001.zip › Figure S3. Cumulative for folate.jpg]

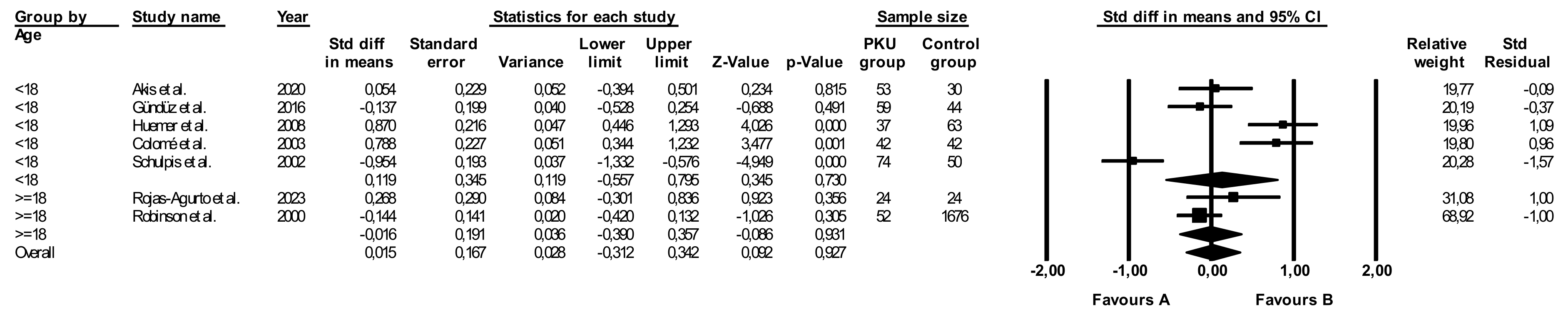

Supplement: Supplementary file 1 [file ijms-25-05065-s001.zip › Figure S30. B12 - age subgroup.jpg]

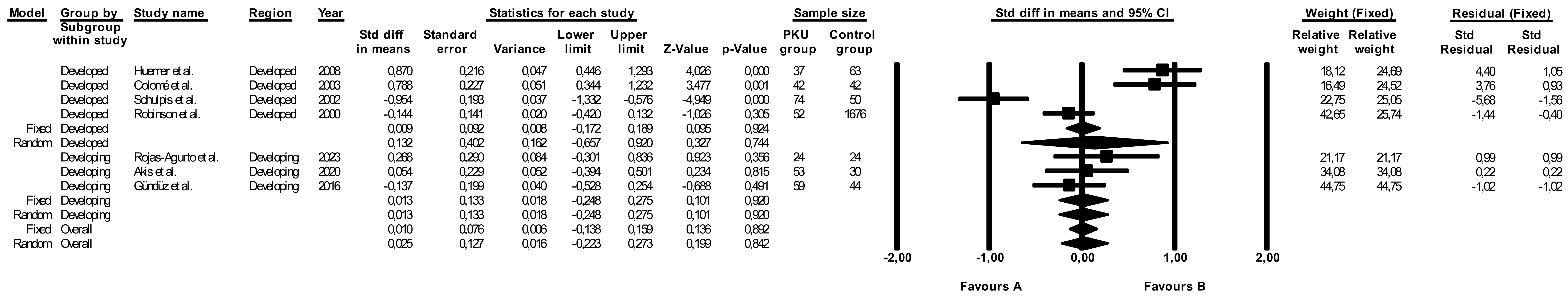

Supplement: Supplementary file 1 [file ijms-25-05065-s001.zip › Figure S31. B12 - country subgroup.jpg]

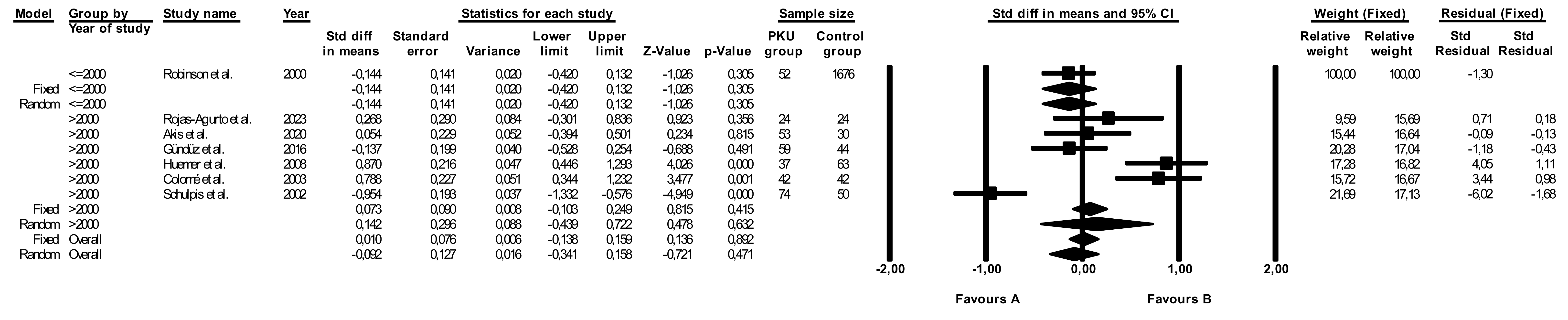

Supplement: Supplementary file 1 [file ijms-25-05065-s001.zip › Figure S32. B12 - year subgroup.jpg]

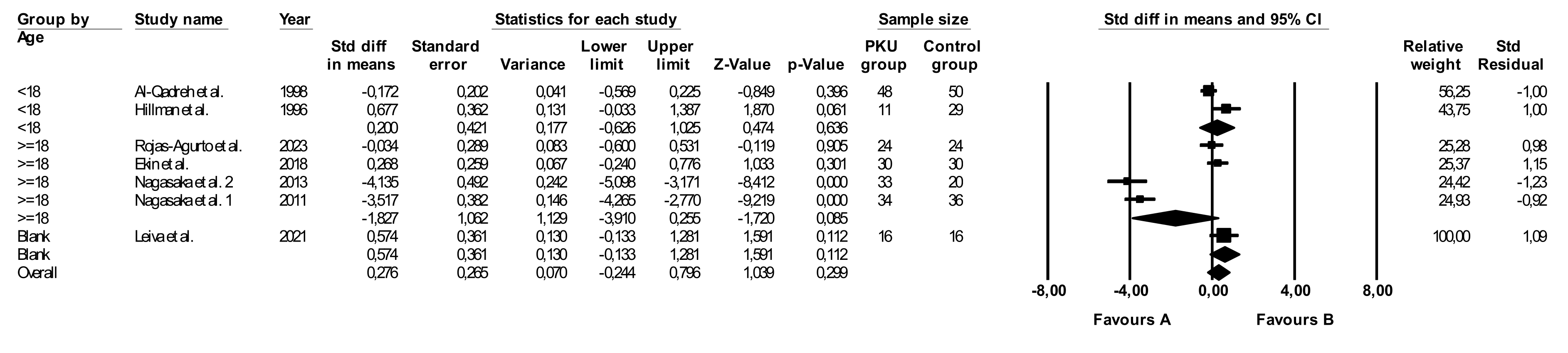

Supplement: Supplementary file 1 [file ijms-25-05065-s001.zip › Figure S33. D - age subgroup.jpg]

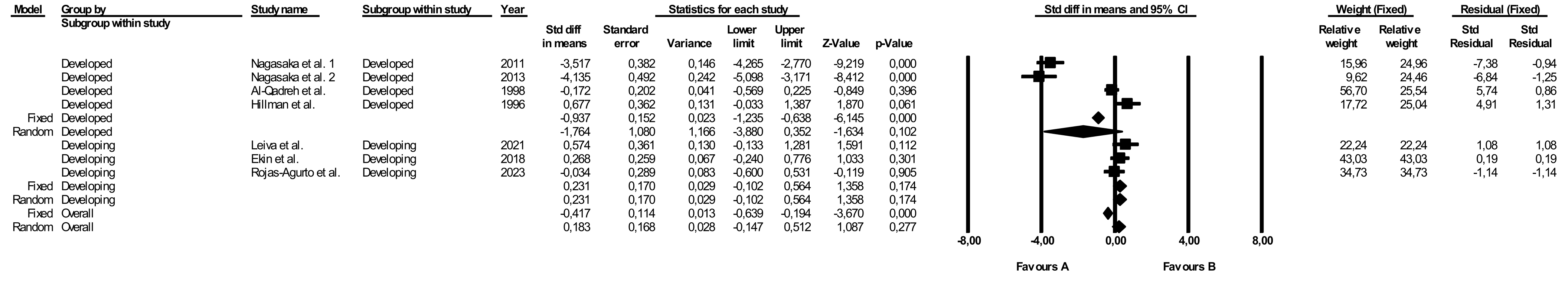

Supplement: Supplementary file 1 [file ijms-25-05065-s001.zip › Figure S34. D - country subgroup.jpg]

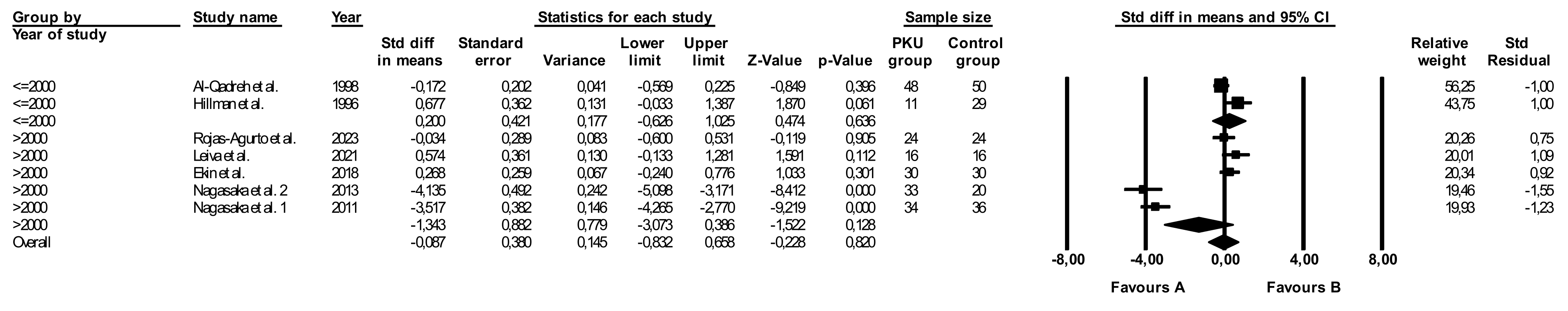

Supplement: Supplementary file 1 [file ijms-25-05065-s001.zip › Figure S35. D - year subgroup.jpg]

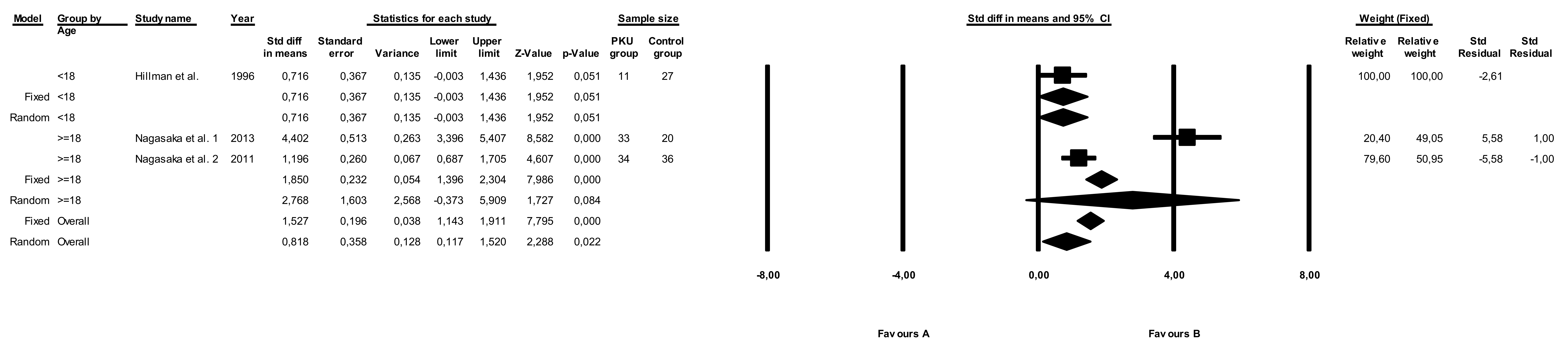

Supplement: Supplementary file 1 [file ijms-25-05065-s001.zip › Figure S36. 1.25D - age subgroup.jpg]

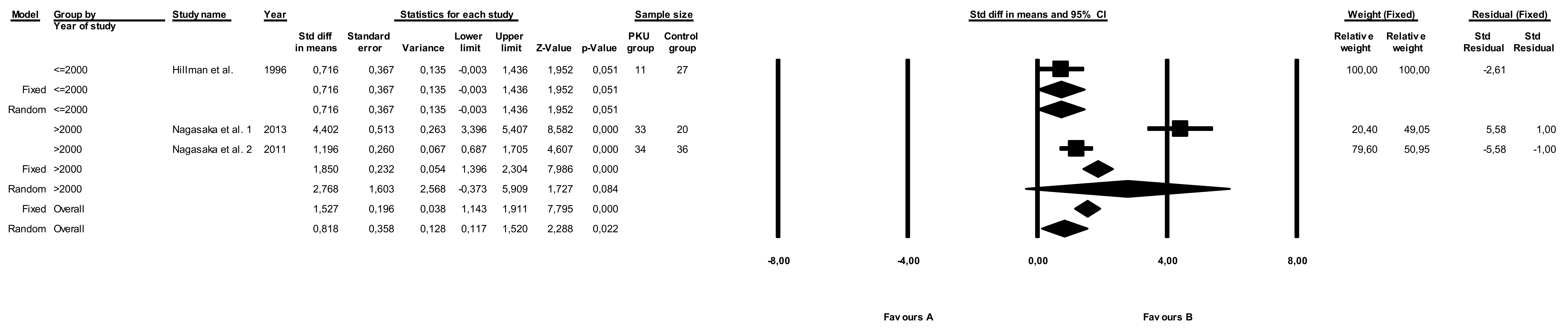

Supplement: Supplementary file 1 [file ijms-25-05065-s001.zip › Figure S37. 1.25D - year subgroup.jpg]

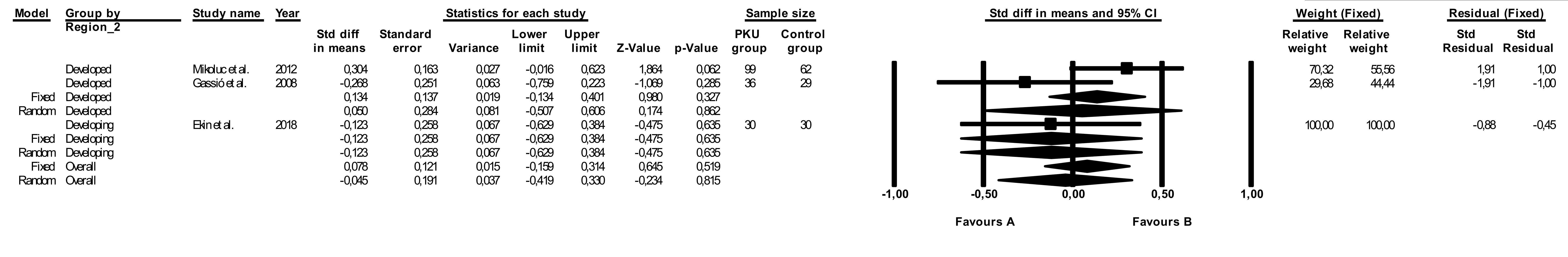

Supplement: Supplementary file 1 [file ijms-25-05065-s001.zip › Figure S38. A - country subgroup.jpg]

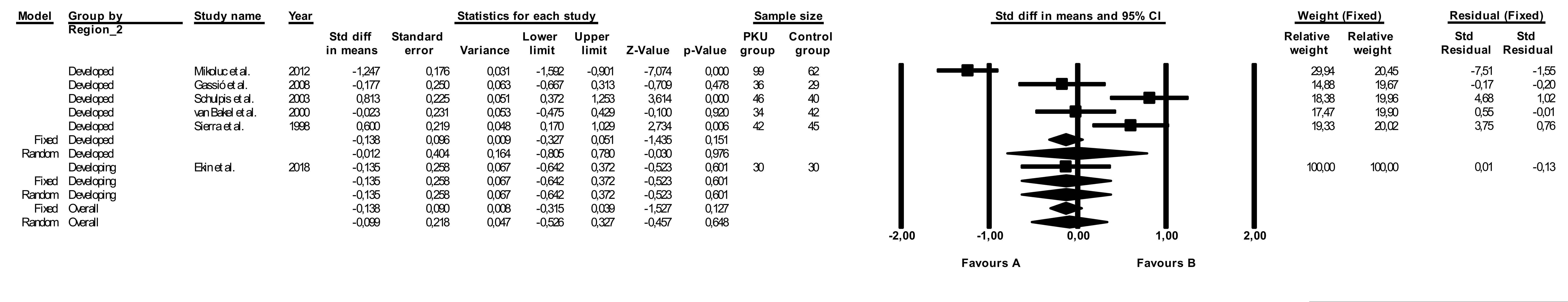

Supplement: Supplementary file 1 [file ijms-25-05065-s001.zip › Figure S39. E - country subgroup.jpg]

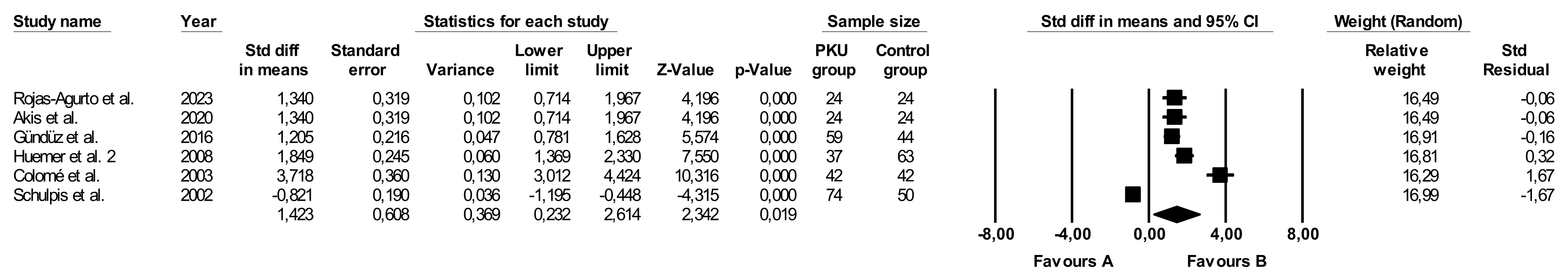

Supplement: Supplementary file 1 [file ijms-25-05065-s001.zip › Figure S4. Folate without hrob.jpg]

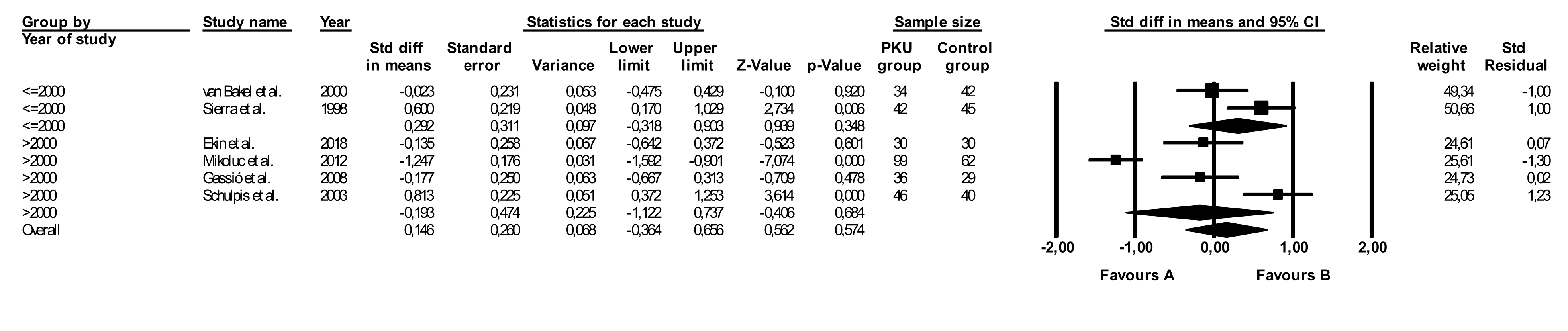

Supplement: Supplementary file 1 [file ijms-25-05065-s001.zip › Figure S40. E - year subgroup.jpg]

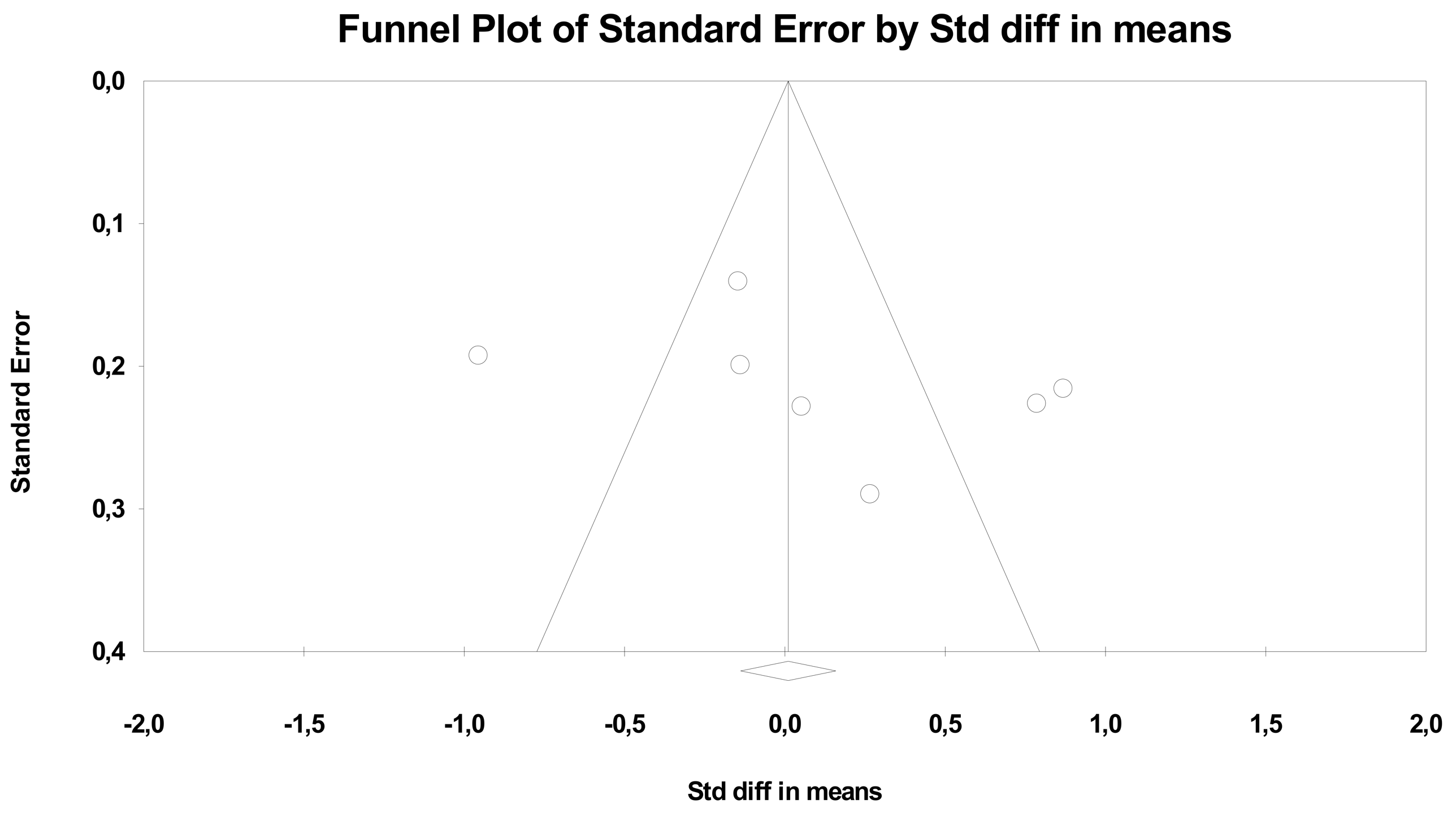

Supplement: Supplementary file 1 [file ijms-25-05065-s001.zip › Figure S5. Funnel plot for B12.jpg]

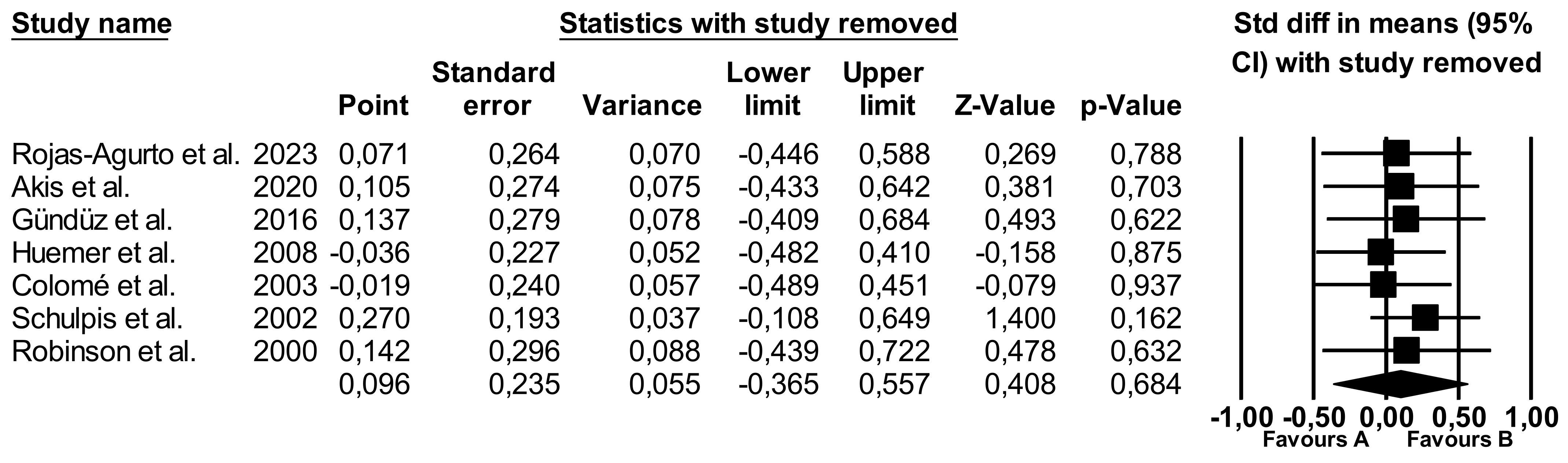

Supplement: Supplementary file 1 [file ijms-25-05065-s001.zip › Figure S6. Sensitivity for B12.jpg]

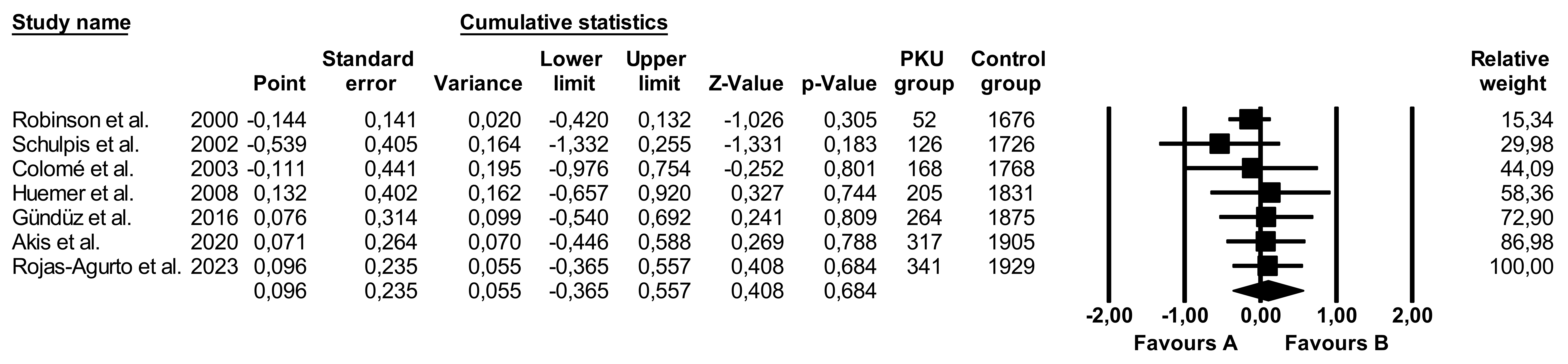

Supplement: Supplementary file 1 [file ijms-25-05065-s001.zip › Figure S7. Cumulative for B12.jpg]

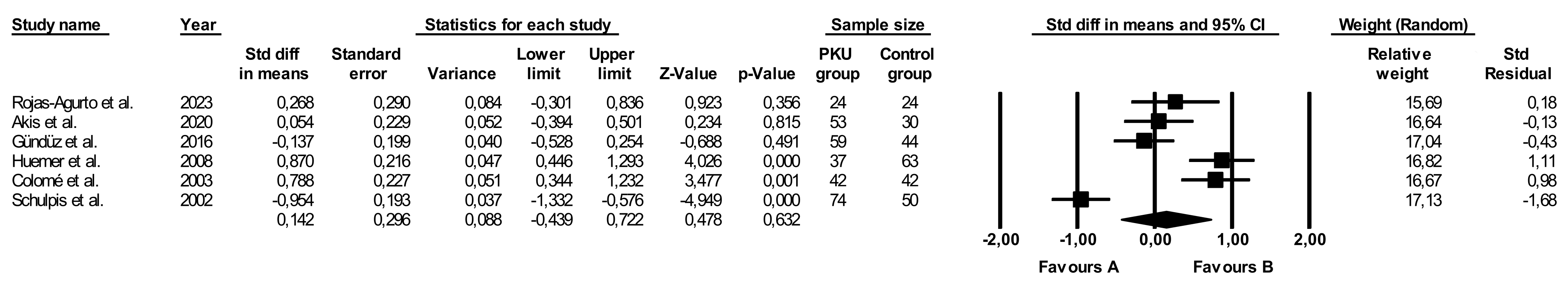

Supplement: Supplementary file 1 [file ijms-25-05065-s001.zip › Figure S8. B12 without hrob.jpg]

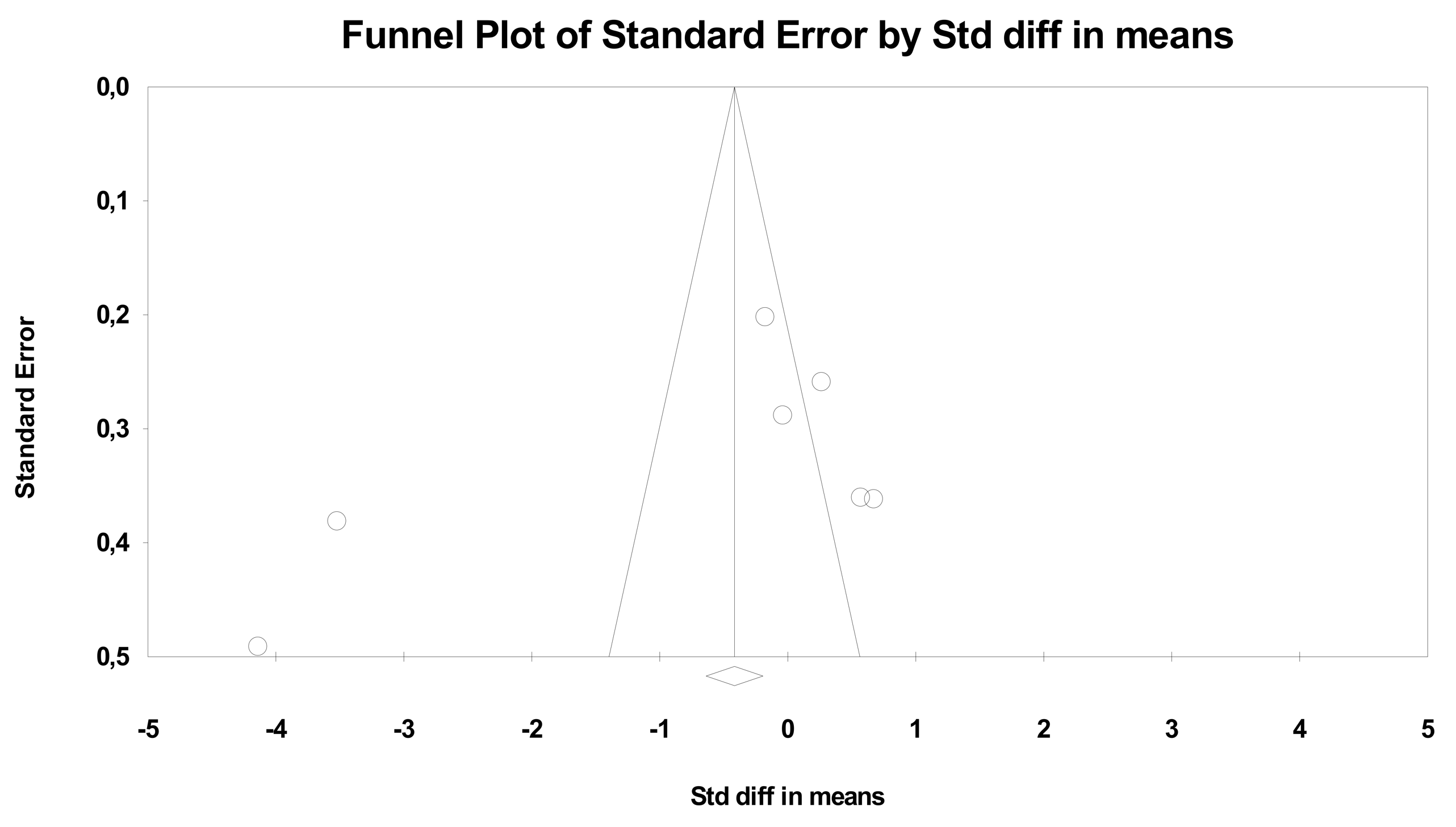

Supplement: Supplementary file 1 [file ijms-25-05065-s001.zip › Figure S9. Funnel plot for vitamin D.jpg]
